# Optimized Protocols for *In-Vitro* T-Cell-Dependent and T-Cell-Independent Activation for B-Cell Differentiation Studies Using Limited Cells

**DOI:** 10.3389/fimmu.2022.815449

**Published:** 2022-06-29

**Authors:** Casper Marsman, Dorit Verhoeven, Jana Koers, Theo Rispens, Anja ten Brinke, S. Marieke van Ham, Taco W. Kuijpers

**Affiliations:** ^1^ Department of Immunopathology, Sanquin Research and Landsteiner Laboratory, University of Amsterdam, Amsterdam, Netherlands; ^2^ Department of Pediatric Immunology, Rheumatology and Infectious Diseases, Emma Children’s Hospital, Amsterdam University Medical Centers, University of Amsterdam, Amsterdam, Netherlands; ^3^ Department of Experimental Immunology, Amsterdam Institute for Infection & Immunity, Amsterdam University Medical Centers (UMC), University of Amsterdam, Amsterdam, Netherlands; ^4^ Swammerdam Institute for Life Sciences, University of Amsterdam, Amsterdam, Netherlands

**Keywords:** B-cell activation, B-cell differentiation, plasma cells, CD40L, IL-21, CpG, IL-2

## Abstract

**Background/Methods:**

For mechanistic studies, *in-vitro* human B-cell differentiation and generation of plasma cells are invaluable techniques. However, the heterogeneity of both T-cell-dependent (TD) and T-cell-independent (TI) stimuli and the disparity of culture conditions used in existing protocols make the interpretation of results challenging. The aim of the present study was to achieve the most optimal B-cell differentiation conditions using isolated CD19^+^ B cells and peripheral blood mononuclear cell (PBMC) cultures. We addressed multiple seeding densities, different durations of culturing, and various combinations of TD and TI stimuli including B-cell receptor (BCR) triggering. B-cell expansion, proliferation, and differentiation were analyzed after 6 and 9 days by measuring B-cell proliferation and expansion, plasmablast and plasma cell formation, and immunoglobulin (Ig) secretion. In addition, these conditions were extrapolated using cryopreserved cells and differentiation potential was compared.

**Results:**

This study demonstrates improved differentiation efficiency after 9 days of culturing for both B-cells and PBMC cultures using CD40L and IL-21 as TD stimuli and 6 days for CpG and IL-2 as TI stimuli. We arrived at optimized protocols requiring 2,500 and 25,000 B–cells per culture well for the TD and TI assays, respectively. The results of the PBMC cultures were highly comparable to the B-cell cultures, which allows dismissal of additional B-cell isolation steps prior to culturing. In these optimized TD conditions, the addition of anti-BCR showed a little effect on phenotypic B-cell differentiation; however, it interferes with Ig secretion measurements. The addition of IL-4 to the TD stimuli showed significantly lower Ig secretion. The addition of BAFF to optimized TI conditions showed enhanced B-cell differentiation and Ig secretion in B-cell but not in PBMC cultures. With this approach, efficient B-cell differentiation and Ig secretion were accomplished when starting from fresh or cryopreserved samples.

**Conclusion:**

Our methodology demonstrates optimized TD and TI stimulation protocols for more in-depth analysis of B-cell differentiation in primary human B-cell and PBMC cultures while requiring low amounts of B cells, making them ideally suited for future clinical and research studies on B-cell differentiation of patient samples from different cohorts of B-cell-mediated diseases.

## Introduction

B-cells are an essential arm of adaptive immunity as their differentiation in response to foreign antigen generates protective antibodies and immunological memory (1). The process of B-cell differentiation into plasmablasts and plasma cells involves profound molecular changes in morphology, phenotype, and gene expression, enabling the cells to produce and secrete large amounts of immunoglobulins (Igs). B-cell differentiation is initiated by activation of B cells by exposure to antigen. Classically, B-cell responses are categorized into two different B-cell responses dependent on the type of antigen, known as T-cell-dependent (TD) and T-cell-independent (TI) responses (1, 2).

In TD B-cell responses, B cells are usually activated by proteinaceous antigens in the secondary lymphoid organs through recognition of their cognate antigen by the B-cell receptor (BCR). Differentiation of B cells in these circumstances requires T-cell help in the form of CD40-CD40L co-stimulation with T-cell-derived cytokines such as IL-4 and IL-21 (3–5). Initially, this process results in the generation of memory B cells, which can rapidly differentiate into high-affinity antibody-producing plasma cells during secondary antigen exposure (6, 7). Secondly, long-lived plasma cells are generated that move to bone marrow niches from where they secrete high-affinity antibodies (8). These two compartments of humoral immunological memory are hallmarks of many vaccination strategies.

In TI B-cell responses, B cells are activated without T-cell help (9). TI antigens include multimeric antigens, like bacterial capsule polysaccharides (PS) and bacterial DNA, which can activate B cells through binding of the BCR and engagement of specific Toll-like receptors (TLRs) such as TLR-4 and TLR-9 (10–13). In addition to this, multiple different cytokines, produced by multiple immune cells, can interact with their respective receptor expressed on B cells and could potentially modulate the response. TI B-cell responses are short-lived and do not result in the selection of affinity-matured antibodies. However, these TI B-cell responses have been shown to result in long-lived antibody production in specific cases (14). Although antibodies are of fundamental importance in the protection against pathogens, aberrant B-cell differentiation may lead to autoimmune diseases when tolerance governed by immunological checkpoints is lost (15).

A major hurdle in the study of *in-vitro* human B-cell differentiation consists of the various methods described to generate *in-vitro* plasmablasts and plasma cells, which often fail to exactly mimic *in-vivo* responses. In these methods, TD and TI antigens are often combined (**Table 1**). While this is an effective method to induce B-cell differentiation *in-vitro* and combining TD and TI antigens could be of great importance to answer specific research questions (e.g., understanding B-cell responses to specific viruses, bacteria, or vaccines using adjuvants), it does not allow the evaluation of separate and distinct routes of B-cell activation, each being characterized by their own antibody output. Optimal conditions are still elusive and there are many determinant factors. Thus, this study was designed to investigate the most optimal B-cell differentiation conditions with regard to several essential factors, i.e., using isolated CD19^+^ B cells or peripheral blood mononuclear cell (PBMC) cultures, multiple seeding densities, different durations of culturing, and various combinations of TD stimuli or TI stimuli. B-cell expansion, proliferation, and differentiation were analyzed by flow cytometry after 6 and 9 days by measuring B-cell numbers, CellTrace Yellow (CTY) dilution, CD27^+^CD38^+^ plasmablast and CD27^+^CD38^+^CD138^+^ plasma cell formation, and immunoglobulin (Ig) secretion in culture supernatants by enzyme-linked immunosorbent assay (ELISA). In addition, these conditions were extrapolated using cryopreserved cells, and the differentiation potentials of cryopreserved and freshly isolated cells were compared. The resulting protocols are one step and minimalistic, ensuring that the results from different labs are comparable.

**Table 1:** Frequently reported B-cell differentiation stimuli for human naive and/or memory B cells in literature.

Stimuli	Target(s)	Concentrations	Reference
Anti-IgM Anti-IgG/IgM F(ab′)_2_ Anti-IgG/IgA/IgM F(ab′)_2_	BCR	2, **5**, 10 µg/ml	(5, 16–22)
BAFF	BAFF-R, BMCA, TACI	75, **100** ng/ml	(19, 23)
αCD40, CD40L	CD40	50, 500 ng/ml 1, 5 µg/ml	(5, 16, 18, 21, 22, 24–26)
CD40L expressing L cells 3T3-CD40L fibroblasts	CD40	Various ratios of B cell:feeder cell	(3, 17, 19, 20)
CpG-ODN 2006	TLR-9	1.0, 2.5, 3.2, 6.0, 10 µg/ml 0.35, **1.0** µM	(16, 18, 19, 21–26)
IFNα	IFNAR	100, 500 U/ml	(20, 24)
IL-2	IL-2R	20, 50, 100 U/ml 25, **50** ng/ml	(3, 5, 20–22, 24, 25)
IL-4	IL-4R	10, **25**, 50, 100, 200 ng/ml	(3, 5, 16, 17, 19, 21, 24, 26)
IL-6	IL-6R	10, 50 ng/ml	(20, 24)
IL-10	IL-10R	25, 50, 200 ng/ml	(5, 19, 21, 24, 26)
IL-15	IL-15R	10 ng/ml	(19, 24)
IL-21	IL-21R	2, 20, **50**, 100 ng/ml	(5, 17–20, 22, 23, 25, 26)

The concentrations used in this study are depicted in bold. Abbreviations: BAFF, B-cell-activating factor; BCMA, B-cell maturation antigen; IFN, interferon; IL, interleukin; ODN, oligodeoxynucleotide; TACI (TNFRSF13B), transmembrane activator and CAML interactor; TLR, toll-like receptor.

## Materials and Methods

### Literature Review

In order to identify stimuli described in previously reported B-cell differentiation protocols, a literature review was carried out using search terms—B-cell culture, B-cell expansion, B-cell stimulation, B-cell activation, human B-cell differentiation, and human plasma cell differentiation—utilizing the NCBI PubMed database (https://www.ncbi.nlm.nih.giv/pubmed). The results of this literature review are summarized in **Table 1**.

### Cell Lines

NIH3T3 fibroblasts expressing human CD40L (3T3-CD40L^+^) (27) were cultured in IMDM (Lonza, Basel 4002, Switzerland) containing 10% of fetal calf serum (FCS) (Serana, 14641 Pessin, Germany), 100 U/ml of penicillin (Invitrogen, through Thermo Fisher, 2665 NN Bleiswijk, The Netherlands), 100 μg/ml of streptomycin (Invitrogen), 2 mM of L-glutamine (Invitrogen), 50 μM of β-mercaptoethanol (Sigma-Aldrich, 3330 AA, Zwijndrecht, The Netherlands), and 500 μg/ml of G418 (Life Technologies, through Thermo Fisher).

### Isolation of PBMCs and B Cells From Human Healthy Donors

Buffy coats of healthy human donors were obtained from Sanquin Blood Supply, 1066CX Amsterdam, the Netherlands. All the healthy donors provided written informed consent in accordance with the protocol of the local institutional review board, the Medical Ethics Committee of Sanquin Blood Supply, and the study conformed to the principles of the Declaration of Helsinki. The mean age of the healthy donors was 41.3 years at the time of blood withdrawal (range 27–61 years; men, *n* = 2; women, *n* = 4). PBMCs were isolated from buffy coats using a Lymphoprep (Axis-Shield PoC AS, Dundee DD2 1XA, Scotland) density gradient. Afterward, from half of the fraction of PBMCs, CD19^+^ B cells were isolated using magnetic Dynabeads and DETACHaBEAD (Thermo Fisher) according to the manufacturer’s instructions. The purity of B cells after isolation and the B-cell compartment distribution at baseline were assessed by flow cytometry. All B-cell isolations had a purity of >92%. Excess cells were resuspended to 20–50 * 10^6^ cells per ml in a culture medium, and a slowly cold freezing medium (80% of DMSO/20% of FCS, Thermo Fisher) was added at a 1:1 ratio. The cell suspension was resuspended and placed in cryovials. Cells were frozen overnight at −80°C in Mr. Frosty and transferred to a cryo-storage the next morning.

### *In-Vitro* PBMC and B-Cell Stimulation Cultures

3T3-CD40L^+^ were harvested, irradiated with 30 Gy, and seeded in the B-cell medium (RPMI 1640, Gibco through Thermo Fisher) without phenol red containing 5% of FCS, 100 U/ml of penicillin, 100 μg/ml of streptomycin, 2 mM of L-glutamine, 50 μM of β-mercaptoethanol, and 20 μg/ml of human apo-transferrin [Sigma-Aldrich; depleted for human IgG with protein G Sepharose (GE Healthcare, 3871MV, Hoevelaken, The Netherlands)] on 96-well flat-bottom plates (Nunc through Thermo Fisher) to allow adherence overnight. 3T3-CD40L^+^ were seeded by adding 100 µl of 0.1 * 10^6^ cells/ml (or 10,000) of cells per well. In some experiments, PBMCs were thawed from cryo-storage and washed with the B-cell medium. PBMCs or B cells were rested at 37°C for 1 h before counting. Then, 50 µl of CD19^+^ B cells at a concentration of 0.005 * 10^6^, 0.05 * 10^6^, or 0.5 * 10^6^ cells/ml (250, 2,500, or 25,000 cells, respectively) were co-cultured in duplicate in the presence or absence of PBMCs with the irradiated 3T3-CD40L^+^ fibroblasts in TD settings or in 96-well U-bottom plates in TI settings. Fifty microliters of stimuli were added as indicated: F(ab′)_2_ fragment goat anti-human IgA/G/M (5 μg/ml; Jackson ImmunoResearch, Ely CB7 4EZ, UK), IL-4 (25 ng/ml; CellGenix, 79107 Freiburg, Germany), IL-21 (50 ng/ml; Peprotech, London W6 8LL, UK), CpG ODN 2006 (1 µM, Invivogen, F-31400 Toulouse, France), IL-2 (50 ng/ml, Miltenyi, 2333ZZ Leiden, the Netherlands Biotec), and B-cell-activating factor (BAFF) (100 ng/ml, R&D (Bio-techne), Abingdon, OX14 3NB, UK) for up to 9 days. After adding the B cells to the wells, the plate was centrifuged for 1 min at 400×*g* to force all the cells onto the 3T3-CD40L^+^ layer. Cryopreserved cells were thawed by agitating the tubes gently in a 37°C water bath until only a small ice clump was left. The cells were transferred to a 50-ml tube and a cold B-cell medium was added dropwise while the tube was constantly agitated.

### CellTrace Yellow Labeling

CD19^+^ B cells or PBMCs were washed with 10 ml of PBS/0.1% of bovine serum albumin (BSA, Sigma-Aldrich) and resuspended to a concentration of 2 × 10^7^ cells/ml in PBS/0.1% of BSA. Cells and 10 µM of CellTrace Yellow (Thermo Fisher Scientific) were mixed at a 1:1 ratio and incubated for 20 min in a 37°C water bath in the dark, vortexing the tube every 5 min to ensure uniform staining. Cells were washed twice using 10× volume of cold culture medium to end labeling. Thereafter, B cells were cultured according to the protocol described above.

### Flow Cytometry

Wells were resuspended and transferred to 96-well V-bottom plates (Nunc). Cells were centrifuged for 2 min at 600×*g*, and the supernatant was transferred to V-bottom plates, sealed with an ELISA sticker, and stored at −20°C. Samples were washed twice with 150 μl of PBS/0.1% of BSA. Cells were stained in a 25 µl staining mix with 1:1,000 LIVE/DEAD Fixable Near-IR Dead Cell Stain kit (Invitrogen) and antibodies diluted in PBS/0.1% of BSA for 20 min at room temperature (RT) in the dark (**Table 2**). The samples were washed 2× with 150 μl of PBS/0.1% of BSA. Finally, the samples were resuspended in a volume of 140 µl, of which 90 µl was measured on an LSR II or FACSymphony flow cytometer. The samples were measured on the LSR II or Symphony and analyzed using FlowJo software. The gating strategy is shown in **Supplementary Figure 1**.

**Table 2:** Antibodies used for flow cytometry.

Ab	Conjugate	Manufacturer	Clone	Catalog No.	Dilution*
CD19	BV510	BD	SJ25-C1	562947	1:100
CD20	PerCP-Cy5.5	BD	L27	332781	1:25
CD27	PE-Cy7	eBioscience	0323	25-0279-42	1:50
CD38	V450	BD	HB7	646851	1:100
CD138	FITC	BD	MI15	561703	1:50
IgG	BUV395	BD	G17-145	564229	1:100
IgM	APC	Biolegend	MHM-88	314510	1:100
CD3	PerCP	BD	SK7	345766	1:20
LIVE/DEAD	APC-Cy7	Invitrogen		L34976	1:1000

*Optimal antibody dilutions as defined for the method and staining procedure used in this paper. As the staining conditions and flow cytometer settings may differ per lab, it is advised that these dilutions are taken as guidelines and that these are validated within each individual lab.

### ELISA of Culture Supernatants

The supernatants of eligible conditions were tested for secreted IgG, IgA, and IgM with a sandwich ELISA using polyclonal rabbit anti-human IgG, IgA, and IgM reagents and a serum protein calibrator (X0908, Dako (Glostrup) via Agilent Technologies, 1186DS Amstelveen, the Netherlands) all from Dako (Glostrup; product numbers A0423, Q0332, and A0425, respectively). The polyclonal rabbit anti-human IgG, IgA, and IgM were diluted in coating buffer to a concentration of 5 μg/ml, then 100 μl was used to coat Nunc MaxiSorp flat-bottom 96-well plates (Thermo Fisher) overnight at 4°C. Plates were washed with PBS/0.05% of Tween-20 and blocked with 100 μl of PBS/1% of BSA (Sigma-Aldrich) for 1 h at RT. Plates were then washed and 100 μl of serum protein calibrator (X0908, Dako, Glostrup) or culture supernatant diluted in HPE buffer (M1940, Sanquin Reagents, 1066CX Amsterdam, the Netherlands reagents) (1:25 for IgG and IgA and 1:30 for IgM) was added to each well and incubated for 1.5 h at RT. Human serum protein low control (X0939, Dako, Glostrup) was used as a reference sample on each plate. Following incubation and washing, 100 μl of detection antibody diluted in blocking buffer was added: poly rabbit anti-human IgG/HRP (1.3 g/L, 1:15,000), IgA/HRP (1.3 g/L, 1:15,000), and IgM/HRP (1.3 g/L, 1:10,000) (Dako, Glostrup; product numbers: P0214, P0215, and P0216, respectively). Plates were washed and developed using TMB (00-4201-56, Invitrogen by Thermo Fisher), stopped using 1 M of H_2_SO_4_ stopping solution, and read using the BioTek microplate reader (450–540 nm) (Synergy HT, Biotek) via Agilent Technologies, 1186DS Amstelveen, the Netherlands, and IgM, IgA, and IgG concentrations were calculated relative to a titration curve of the serum protein calibrator.

### Interference ELISA

The interference ELISA assay was developed as described in the sandwich ELISA above. Serial dilutions of F(ab′)_2_ fragment goat anti-human IgA/G/M (5, 2.5, 1.25, and 0.625 μg/ml; Jackson ImmunoResearch, Ely CB7 4EZ, UK) in HPE buffer (M1940, Sanquin reagents) were incubated (60 min, RT) with the standard curve dilutions of the serum protein calibrator (X0908, Dako, Glostrup). The results were plotted as titration curves.

### Graphics

Schematic overviews were created using images from Servier Medical Art, which are licensed under a Creative Commons Attribution 3.0 Unported License (http://smart.servier.com).

### Statistical Analysis

Statistical analysis was performed using GraphPad Prism (version 8; GraphPad software). Data were analyzed using *t*-tests, repeated measures one-way ANOVA, or repeated measures two-way ANOVA where appropriate. Results were considered significant at *P <*0.05. Significance was depicted as * (*P* < 0.05), ** (*P* < 0.01), *** (*P* < 0.001), or **** (*P* < 0.0001). Correlation analyses between flow cytometry and immunoglobulin ELISA data were performed with Pearson’s correlation test (*P < 0.05). The statistical tests performed are indicated in the figure legends.

## Results

### Frequently Reported B-Cell Differentiation Stimuli for Human Naive and/or Memory B Cells in the Literature

The first step in establishing optimized *in-vitro* protocols for TD and TI stimulation of B cells to induce B-cell differentiation was to identify frequently used and reported culture conditions by a literature review (**Table 1**). Following the identification of a wide range of stimuli, together with consortium partner labs, standard TD and TI combinations were chosen using concentrations reflective of the publications obtained through literature review or by previous experimental work. For the TD stimuli, the combination of CD40L and IL-21 was selected, a combination frequently used to mimic CD4^+^ T-cell help (5). Although multiple methods of CD40L stimulation were reported (either soluble or using feeder cells), here a monolayer of feeder cells consisting of 3T3 mouse fibroblast expressing high levels of human CD40L was selected. For TI, the combination of CpG, a well-known ligand of TLR-9, together with IL-2, a B-cell survival factor, was set up (16, 28). These combinations of stimuli were either constantly or most frequently used and therefore found to be essential.

Following the identification of CD40L and IL-21 as TD stimuli and CpG and IL-2 as TI stimuli, the effect of 1) culture duration and 2) different seeding densities (or starting B-cell numbers) was determined. These experiments were performed with cells from healthy donors either using 3) purified CD19^+^ B cells or 4) PBMC cultures corrected for B-cell count, comprised of B cells and other PBMCs (mainly T cells and small fractions of monocytes and NK cells), since such cultures do not require B-cell isolation and are thus more practical for routine use. For this, PBMCs from the same healthy donors were used to avoid donor variability. Additionally, the augmenting effect of 5) additional stimuli, i.e., anti-BCR and IL-4 for TD and anti-BCR and BAFF for TI, was investigated, and 6) the effect of cryopreservation on B-cell differentiation potential was checked. All conditions were cultured in duplicate.

### Efficient *In-Vitro* B-Cell Differentiation After 9 Days Using T-Cell-Dependent Stimulation With CD40L and IL-21 Using 2,500 Starting B Cells

**Figure 1:**
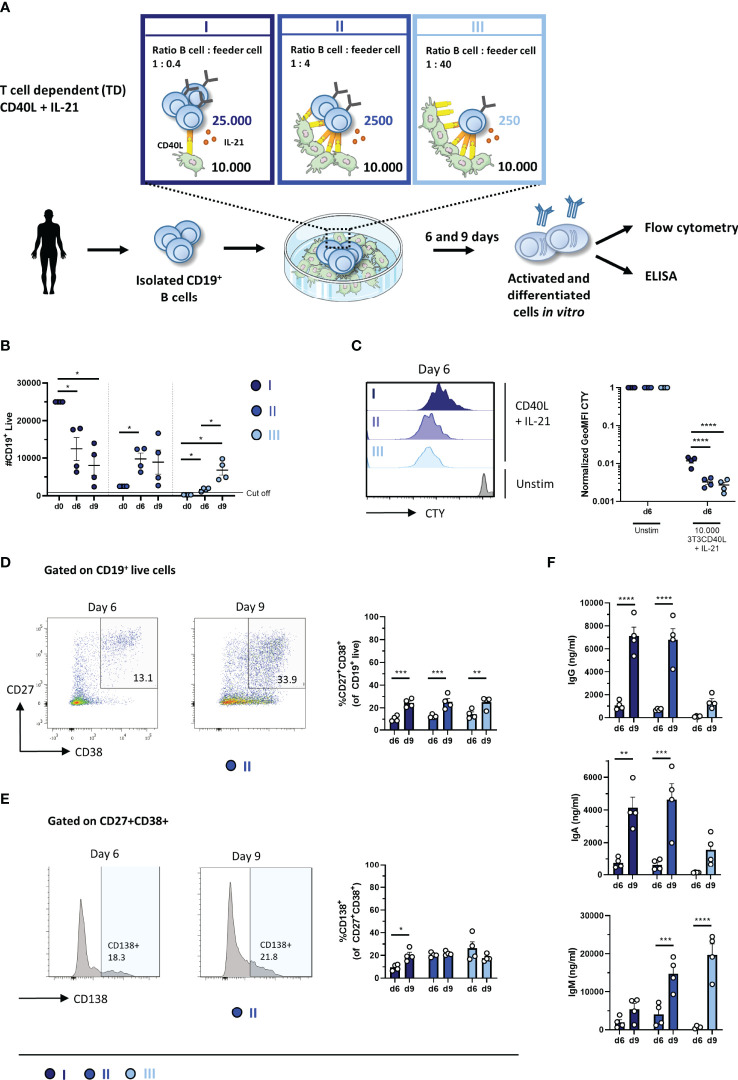
Proliferation, differentiation, and antibody production after T-cell-dependent *in-vitro* stimulation and culturing of low numbers of primary human CD19^+^ B cells. **(A)** Schematic overview of the T-cell-dependent (TD) culture system to induce B-cell differentiation. A total of 25,000, 2,500, or 250 CD19^+^ human B cells (*n* = 4) were stimulated with a human CD40L-expressing 3T3 feeder layer and recombinant IL-21 (50 ng/ml) enabling conditions I (dark blue), II (cobalt blue), and III (light blue). Cells were analyzed on day 6 and day 9 by flow cytometry to evaluate the **(B)** number of live CD19^+^ events, **(C)** amount of proliferation by CTY dilution, and frequency of **(D)** plasmablast (CD27^+^CD38^+^ B cells) and **(E)** plasma cell (CD27^+^CD38^+^CD138^+^ B cells). A cutoff of 1,000 events was used to proceed with further analysis. **(F)** The supernatant was collected on day 6 and day 9 to evaluate IgG, IgA, and IgM production by ELISA (*n* = 4). Each data point represents the mean of an individual donor with duplicate culture measurements. Mean values are represented by bars and the error bars depict SEM. *P*-values were calculated using two-way ANOVA with Sidak’s multiple comparison test. *P ≤ 0.05, **P ≤ 0.01, ***P ≤ 0.001, ****P ≤ 0.0001.

In the TD assay, either 25,000, 2,500, or 250 starting B cells were co-cultured with CD40L feeder cells and IL-21 enabling three conditions, hereupon referred to as conditions I, II, and III (**Figure 1A**). Due to these settings, different ratios of B cell to feeder cell (1:0.4, 1:4, and 1:40, respectively) were created, resulting in different availability of CD40L during culturing. For each condition, B-cell expansion and proliferation were assessed by flow cytometry after 6 and 9 days as well as plasmablast (CD27^+^CD38^+^) and plasma cell (CD27^+^CD38^+^CD138^+^) formation (for the gating strategy, see **Supplementary Figure 1**). Additionally, the secretion of IgG, IgA, and IgM was measured by ELISA in culture supernatants, which acts as a second readout for B-cell differentiation as B cells differentiate from surface Ig-expressing cells to Ig-secreting cells.

To assess the expansion of B cells during culturing, the number of CD19^+^ live B cells was determined. Conditions II and III showed significantly more CD19^+^ live B cells compared to its specific starting B-cell numbers on day 6 and day 9, while a significant decline in CD19^+^ live B cells in condition I was observed (**Figure 1B**). Condition II showed a 4-fold amplification ( ± 0.6; *n* = 4) on day 6 and a 4-fold amplification ( ± 1.3; *n* = 4) on day 9 compared to its starting B-cell number (**Table 3**). Condition III showed a 7-fold amplification ( ± 0.9; *n* = 4) on day 6 and a 27-fold amplification ( ± 5.2; *n* = 4) on day 9. Interestingly, in all three conditions, a similar yield of CD19^+^ live B cells was detected on day 9, but not on day 6, while the starting B-cell numbers were different, i.e., 10–100-fold. As shown before, this suggests that the amount of available CD40L critically influences B-cell survival and/or expansion during culture (17). For further FACS analysis, we set a cutoff value at a minimum of 1,000 events of CD19^+^ live B cells. To assess proliferation, B cells were labeled with CTY prior to culturing. Proliferation was observed in all conditions on day 6 (**Figure 1C**); however, conditions II and III showed a significantly higher dilution of CTY in accordance with the amplification in cell numbers observed. A significant increase in the percentage of CD27^+^CD38^+^ cells between day 6 and day 9 in each condition was observed, suggesting that a 9-day culture period induced higher levels of differentiation (**Figure 1D**). However, we did not observe differences in plasma cell formation between day 6 and day 9 (**Figure 1E**), and there was no significant difference between the three culture conditions (statistics not shown). Measurement of secreted IgG, IgA, and IgM showed a significant increase between day 6 and day 9, confirming that a 9-day culture period induced higher levels of differentiation and Ig secretion (**Figure 1F**). Notably, although the yield of CD19^+^ live B cells and phenotypically differentiated plasmablasts and plasma cells was comparable in each condition on day 9, different Ig secretion patterns between the three conditions were observed. When we compared the three conditions in the flow cytometry analysis, significantly higher percentages of CD27^−^CD38^+^ B cells were observed in conditions II and III (**Supplementary Figures 2A, B**).

**Table 3:** B cell survival and proliferation during different B cell differentiation conditions.

Isolated CD19^+^ B cells (TD)						
	Condition I		Condition II		Condition III	
Starting B cell number	25.000		2500		250	
3T3-CD40L cell	10.000		10.000		10.000	
Ratio B cell : 3T3-CD40L cell	1 : 0.4		1 : 4		1 : 40	
Cytokines	IL-21		IL-21		IL-21	
Mean CD19^+^ live cells						
Day 0	25.000		2500		250	
Day 6	12438	± 3120; n=4	9736	± 1566; n=4	1656	± 224; n=4
Day 9	8012	± 2643; n=4	8899	± 3196; n=4	6769	± 1305; n=4
Mean amplification (compared to starting B cell number)						
Day 6	0.5	± 0.1; n=4	3.9	± 0.6; n=4	6.6	± 0.9; n=4
Day 9	0.3	± 0.1; n=4	3.6	± 1.3; n=4	27.1	± 5.2; n=4
**PBMCs (TD)**						
	**Condition I.2**		**Condition II.2**		**Condition III.2**	
Starting B cell number	25.000		2500		250	
3T3-CD40L cell	10.000		10.000		10.000	
Ratio B cell : 3T3-CD40L cell	1 : 0.4		1 : 4		1 : 40	
Cytokines	IL-21		IL-21		IL-21	
Mean CD19^+^ live cells						
Day 0	25.000		2500		250	
Day 6	8601	± 1397; n=3	14487	± 4320; n=3	4139	± 311; n=3
Day 9	6019	± 695; n=3	7819	± 2713; n=3	12826	± 3750; n=3
Mean amplification (compared to starting B cell number)						
Day 6	0.3	± 0.1; n=3	5.8	± 1.7; n=3	16.6	-
Day 9	0.2	± 0.1; n=3	4.2	± 1.1; n=3	51.3	-
**Isolated CD19^+^ B cells (TI)**						
	**Condition IV**		**Condition V**		**Condition VI**	
Starting B cell number	25.000		2500		250	
TLR ligand	CpG		CpG		CpG	
Cytokines	IL-2		IL-2		IL-2	
Mean CD19^+^ live cells						
Day 0	25.000		2500		250	
Day 6	4751	± 1397; n=4	85	± 22.7; n=4	17	± 2.3; n=4
Day 9	1571	± 695; n=4	29	± 13.8; n=4	2	± 0.8; n=4
Mean amplification (compared to starting B cell number)						
Day 6	0.2	± 0.1; n=4	-	-	-	-
Day 9	0.1	± 0.0; n=4	-	-	-	-
**PBMCs (TI)**						
	**Condition IV.2**		**Condition V.2**		**Condition VI.2**	
Starting B cell number	25.000		2500		250	
TLR ligand	CpG		CpG		CpG	
Cytokines	IL-2		IL-2		IL-2	
Mean CD19^+^ live cells						
Day 0	25.000		2500		250	
Day 6	8978	± 1985; n=3	3823	± 1983; n=3	58	± 18; n=3
Day 9	7841	± 2127; n=3	2463	± 1055; n=3	47	± 26; n=3
Mean amplification (compared to starting B cell number)						
Day 6	0.4	± 0.1; n=3	1.5	± 0.8; n=3	-	-
Day 9	0.3	± 0.1; n=3	1.0	± 0.4; n=3	-	-

Purified B cells or non-purified B cells (PBMC cultures) were cultured using a 1-step culture system for 6 and 9 days with either TD (CD40L + IL-21) or TI (CpG + IL-2) stimuli. On day 6 and day 9 cell counts and viability were determined using flow cytometry with fluorochrome-conjugated Live/Dead and anti-CD19. Results are shown as the mean ± SEM of n = 4 (B cell cultures) or n = 3 (PBMC cultures) donors. – indicates no further analysis due to <1000 CD19^+^ live events.

In parallel experiments, PBMCs (corrected for 250, 2,500, and 25,000 B cells) were cultured in similar conditions, which created conditions I.2, II.2, and III.2 (**Supplementary Figure 3A**). Here, we observed again significantly more B-cell expansion and proliferation in conditions II.2 and III.2 with higher CD40L availability (**Supplementary Figures 3B, C** and **Table 3**). The number of CD19^+^ live cells and dilution of CTY showed similar results as in conditions I, II, and III suggesting a limited effect of the presence of PBMCs with CD40L and IL-21 stimulation. Proliferation analysis of CD3^+^ T cells did not show any increased proliferation compared to unstimulated controls, indicating that the used stimuli did not activate T cells which would influence B-cell differentiation (data not shown). Again, there was no significant difference between the three conditions (statistics not shown) (**Supplementary Figure 3D**). No effect was observed regarding the presence of PBMCs on the efficacy of plasmablasts or plasma cell induction (**Figures 1D–E** and **Supplementary Figures 3D, E**). Ig measurements in the supernatants of the PBMC cultures showed a significant increase in IgA and IgM secretion between days 6 and 9, indicating that a 9-day culture period induced higher levels of differentiation and Ig secretion (**Supplementary Figure 3F**).

Taken together, conditions I, II, and III with 250, 2,500, and 25,000 starting B cells, respectively, were all suitable for generating CD27^+^CD38^+^ plasmablasts and IgG secretion on day 9, two important hallmarks of B-cell differentiation. The optimal differentiation conditions for TD stimulation with limited numbers of B cells were defined here as conditions II and II.2, being 2,500 CD19^+^ cells per 96-well with or without other PBMCs, which were used for further experiments.

### CD40L and IL-21 Stimulation in Combination With Anti-BCR and/or IL-4 Does Not Increase B-Cell Differentiation and Immunoglobulin Secretion

**Figure 2:**
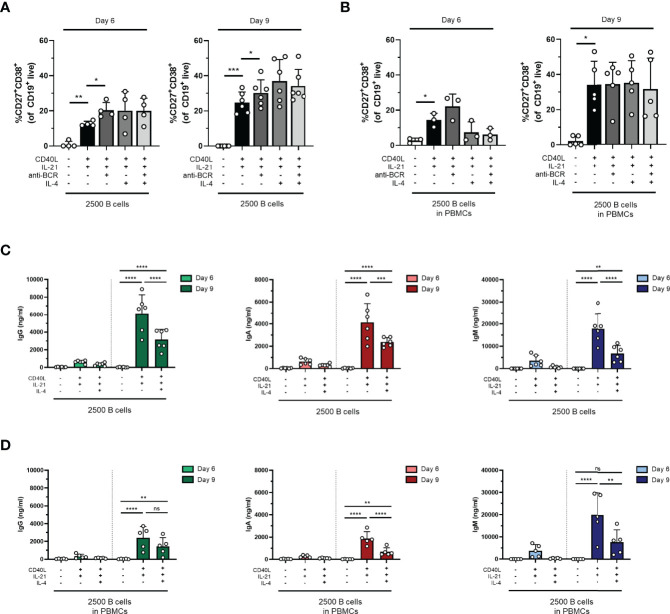
The addition of anti-BCR stimuli in a T-cell-dependent stimulation results in increased B-cell differentiation, while IL-4 severely inhibits antibody production. Human primary B cells obtained from healthy donors were stimulated under conditions described in **Figure 1A** (condition II) and **Supplementary Figure 2A** (condition II.2, PBMC cultures) with or without anti-BCR [anti-Ig F(ab)_2_ mix (5 µg/ml) targeting IgM, IgG, and IgA] and/or recombinant IL-4 (25 ng/ml). Frequencies of CD27^+^CD38^+^ B cells on day 6 and day 9 in **(A)** condition II and **(B)** condition II.2 (*n* = 4–6). **(C, D)** Total secretion of IgG, IgA, and IgM measured in culture supernatants of eligible conditions after 6 and 9 days **(C)** without PBMCs (condition II) and **(D)** within PBMC cultures (condition II.2). Each data point represents the mean of an individual donor with duplicate culture measurements. Mean values are represented by bars and the error bars depict SEM. *P*-values were calculated using one-way ANOVA with Dunnett’s multiple comparison test **(A, B)** or two-way ANOVA with Sidak’s multiple comparison test **(C, D)**. *P ≤ 0.05, **P ≤ 0.01, ***P ≤ 0.001, ****P ≤ 0.0001. ns, not significant.

In an attempt to drive differentiation and expansion even further in our one-step *in-vitro* B-cell differentiation assay, the effect of additional stimuli in our culture conditions was tested. For this purpose, the reference stimuli CD40L and IL-21 were combined with or without F(ab)_2_ fragments targeting IgM, IgG, and IgA to induce BCR signaling (also referred to as anti-BCR). Secondly, we tested whether the addition of IL-4, a cytokine important for naive B cells during the GC reactions, can augment *in-vitro* B-cell differentiation induced by CD40L and IL-21. In these cultures, 2,500 freshly isolated CD19^+^ B cells (condition II) or PBMCs corrected for B-cell number (2,500 B cells; condition II.2) were used from the same donors shown in the previous experiments. Flow cytometry was performed on day 6 and day 9 to classify CD19^+^ cells as CD27^+^CD38^+^ plasmablasts, and the secretion of IgG, IgA, and IgM was measured in the culture supernatants. In condition II, we observed a significant increase in plasmablasts upon adding anti-BCR both on day 6 and day 9 compared to CD40L and IL-21 alone (**Figure 2A**). In condition II.2, no combination of stimuli was superior to CD40L and IL-21 (**Figure 2B**). Prolonged culture to 9 days allowed a significant increase in plasmablasts in all four combinations of stimuli both in conditions II and II.2 (statistics not shown). As the addition of anti-BCR and/or IL-4 stimulation to conditions I, II, or III (and PBMC culture variants of these) could also affect the proliferation of CD19^+^ cells, this was investigated, showing no significant differences (**Supplementary Figures 4A, B**). In the main condition with 2,500 cells, significantly fewer cells were found when adding IL-4 to isolated CD19^+^ cultures on day 6 but not on day 9, or in PBMC cultures (**Supplementary Figures 4C, D**). As the use of the mixture of F(ab)_2_ fragments in our cultures might interfere with the IgG, IgA, and IgM ELISA assay, an interference ELISA was performed. Indeed, we observed a decrease in measured IgG, IgA, and IgM when F(ab)_2_ fragments in different concentrations were added to the standard curve (**Supplementary Figures 5A–C**). Therefore, samples containing anti-BCR stimulation were excluded for further analysis of secreted Ig. Although the percentages of plasmablasts on day 9 were similar (or higher) upon the addition of IL-4, we observed significantly lower secreted IgG, IgA, and IgM in culture supernatants in the conditions where IL-4 was added (**Figures 2C, D**). In conclusion, an augmenting effect of anti-BCR on TD-induced B-cell differentiation was found, but its use prevented Ig secretion analysis. Notably, although we observed no significant effect on plasmablast differentiation, IL-4 reduced Ig secretion under all conditions tested.

### Efficient *In-Vitro* B-Cell Differentiation After 6 Days Using T-Cell-Independent Stimulation With CpG and IL-2 With 25,000 Starting B Cells

**Figure 3:**
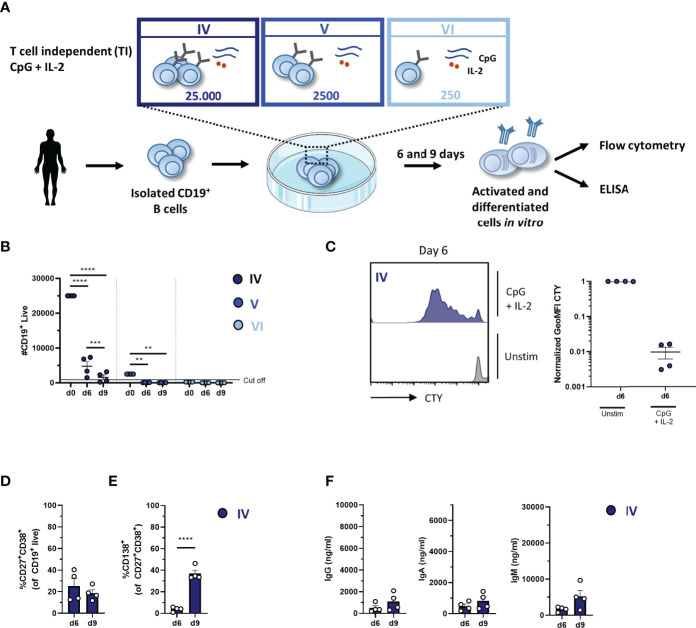
Proliferation, differentiation, and antibody production after T-cell-independent *in-vitro* stimulation and culturing of low numbers of primary human CD19^+^ B cells. **(A)** Schematic overview of the T-cell-independent (TI) culture system to induce B-cell differentiation. A total of 25,000, 2,500, or 250 CD19^+^ human B cells (*n* = 4) were stimulated with CpG (1 µM) and IL-2 (50 ng/ml) enabling conditions IV (dark blue), V (cobalt blue), and VI (light blue). Cells were analyzed on day 6 and day 9 by flow cytometry to evaluate the **(B)** number of live CD19^+^ events, **(C)** amount of proliferation by CTY dilution, and frequency of **(D)** plasmablast (CD27^+^CD38^+^) and **(E)** plasma cell (CD27^+^CD38^+^CD138^+^) generation. A cutoff of 1,000 events was used to proceed with further analysis. **(F)** The supernatant was collected on day 6 and day 9 to evaluate IgG, IgA, and IgM production by ELISA (*n* = 4). Each data point represents the mean of an individual donor with duplicate culture measurements. Mean values are represented by bars and the error bars depict SEM. *P*-values were calculated using two-way ANOVA with Sidak’s multiple comparison test **(B)** or unpaired *t*-test **(D–F)**. **P ≤ 0.01, ***P ≤ 0.001, ****P ≤ 0.0001.

In the TI assay, the effect of 1) culture duration and 2) different seeding densities (or starting B-cell numbers) was also determined. Again 25,000, 2,500, or 250 CD19^+^ B cells were cultured, enabling conditions IV, V, and VI (**Figure 3A**). We assessed B-cell differentiation by flow cytometry analysis and measurements of Ig secretion on day 6 and day 9. Culturing CD19^+^ B cells with TI stimuli resulted in a decline in CD19^+^ B cells (**Figure 3B** and **Table 3**). Flow cytometry analysis of conditions V and VI showed less than 1,000 events on day 6 and day 9, and these conditions were therefore excluded from further analysis. In condition IV, a significant decline in CD19^+^ live cells was observed on day 9 compared to day 6, with two out of four donors not meeting the cutoff of 1,000 events; thus, longer culture periods under TI conditions resulted in lower B-cell survival and/or expansion. Samples eligible for further flow cytometry analysis showed sufficient proliferation on day 6 (**Figure 3C**). There was no significant difference between day 6 and day 9 in terms of CD27^+^CD38^+^ plasmablasts, but there was a significant increase in CD27^+^CD38^+^CD138^+^ plasma cells on day 9 (**Figures 3D, E**). Accordingly, a small increase of secreted IgG, IgA, and IgM in culture supernatants was observed on day 9 (**Figure 3F**).

Extrapolation of the TI conditions to PBMC cultures enabled conditions IV.2, V.2, and VI.2 (**Supplementary Figure 6A**). Consistent with condition IV, we observed a significant decrease in CD19^+^ live B cells in condition IV.2 (**Supplementary Figure 6B**). Interestingly, we observed 1.0- and 1.5-fold ( ± 0.8, *n* = 3; ± 0.4, *n* = 3) amplification of B-cell numbers on days 6 and 9 in condition V.2, which provided sufficient B-cell numbers for further analysis (in contrast to condition V) (**Supplementary Figure 6B** and **Table 3**). As shown before, this suggested an additional pro-survival effect of PBMCs in these cultures (16). Condition VI.2 did not meet the cutoff of 1,000 events and was also excluded from further analysis. Proliferation analysis by CTY dilution showed a significant difference in the proliferation of CD19^+^ live B cells on day 6 between conditions IV.2 and V.2 (**Supplementary Figure 6C**). Proliferation analysis of CD3^+^ T cells showed minimal proliferation compared to unstimulated controls in conditions IV.2 and V.2, which suggested that the used stimuli (likely IL-2) could activate the T cells in these PBMC cultures, possibly influencing B-cell differentiation (**Supplementary Figure 6D**). Further analysis showed no significant difference between day 6 and day 9 in terms of plasmablasts and plasma cells (**Supplementary Figures 6E, F**). Despite the lack of increase in the percentages of CD27^+^CD38^+^ plasmablasts and CD27^+^CD38^+^ CD138^+^ plasma cells between day 6 and day 9, a small increase of secreted IgG, IgA, and IgM was observed in culture supernatants on day 9 in conditions IV.2 and V.2 (**Supplementary Figure 6G**). These experiments identified condition IV and condition IV.2, being 25,000 CD19^+^ cells per well, as the most suitable for the assessment of B-cell differentiation using TI stimulation.

### CpG- and IL-2-Induced B-Cell Differentiation Can Be Amplified by Anti-BCR and BAFF Stimulation

**Figure 4:**
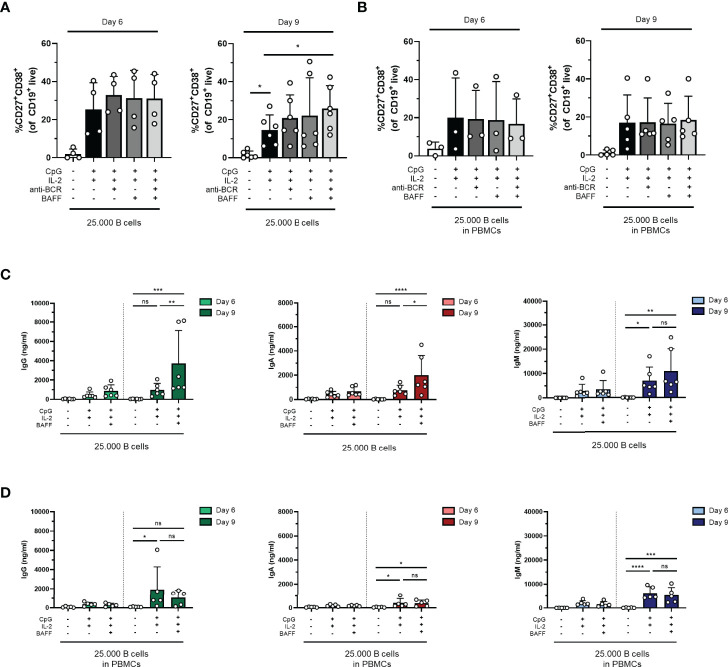
The addition of BAFF in a T-cell-independent stimulation results in increased IgG and IgA production in isolated B-cell cultures. Human primary B cells obtained from healthy donors were stimulated under conditions described in **Figure 3A** (condition IV) and **Supplementary Figure 4A** (condition IV.2, PBMC cultures) with or without anti-BCR [anti-Ig F(ab)_2_ mix (5 µg/ml) targeting IgM, IgG, and IgA] and/or BAFF (100 ng/ml). Frequencies of CD27^+^CD38^+^ B cells on day 6 and day 9 in **(A)** condition IV and **(B)** condition IV.2 (*n* = 3–5). **(C, D)** Total secretion of IgG, IgA, and IgM measured in culture supernatants of eligible conditions after 6 and 9 days **(C)** without PBMCs (condition IV) and **(D)** as PBMC culture (condition IV.2). Each data point represents the mean of an individual donor with duplicate culture measurements. Mean values are represented by bars and the error bars depict SEM. *P*-values were calculated using one-way ANOVA with Dunnett’s multiple comparison test **(A, B)** or two-way ANOVA with Sidak’s multiple comparison test **(C, D)**. *P ≤ 0.05, **P ≤ 0.01, ***P ≤ 0.001, ****P ≤ 0.0001. ns, not significant.

To assess the potential of further assay optimization, the effect of additional stimuli to augment TI-induced differentiation was investigated. For this purpose, anti-BCR stimulation with or without BAFF, another well-known survival factor and differentiation signal for B cells, was supplemented to the reference stimuli CpG and IL-2. Studies primarily done *in-vitro* have shown that BAFF can be expressed by different immune cell types (including monocytes, macrophages, and follicular dendritic cells); however, the contribution of BAFF-producing cells to specific B-cell responses *in vivo* is not yet understood (29–31). In these cultures, 25,000 freshly isolated CD19^+^ B cells (condition IV) or PBMCs corrected for B-cell number (25,000 B cells; condition IV.2) were used from the same donors shown in the previous experiments. We observed a small increase in plasmablasts upon the addition of anti-BCR and/or BAFF both on day 6 and day 9 in condition IV but not in condition IV.2, suggesting that anti-BCR, BAFF, or a combination of both can amplify plasmablast formation in the cultures without the presence of other PBMCs (**Figures 4A, B**). Prolonged culture to 9 days did not result in higher percentages of plasmablasts in any four combinations of stimuli both in conditions IV and IV.2 (statistics not shown). In condition IV, analysis of the samples without anti-BCR stimulation showed that the addition of BAFF resulted in significantly higher secreted IgG, IgA, and IgM in the culture supernatants, while this effect was not present in condition IV.2, where other PBMCs were present (**Figures 4C, D**). As the addition of anti-BCR and/or BAFF stimulation to conditions IV, V, or VI (and PBMC culture variants of these) could also affect the proliferation of CD19^+^ cells, this was investigated, showing no significant differences (**Supplementary Figures 7A, B**). In the main condition with 2,500 cells, significantly more cells were found when anti-BCR stimulation was added to isolated CD19^+^ cultures on day 6 but not on day 9 (**Supplementary Figure 7C**). In PBMC cultures, there was a trend toward higher CD19^+^ counts when BAFF was present although this was not significant (**Supplementary Figure 7D**). Taken together, the addition of BAFF stimulation to CpG and IL-2 augmented TI-induced differentiation and Ig secretion upon culturing purified B cells, but this effect was absent in PBMC cultures.

### Proportion of CD27-Expressing and Class-Switched Cells at the Start of Culture Correlated With *In-Vitro*-Induced Differentiation and Ig Secretion

The ratio of naive to memory cells or the proportion of IgM or Ig-switched cells at the start of culture could be a determining factor for both TD and TI culture results. Thus, we investigated if specific B-cell memory or Ig subsets on day 0 (**Supplementary Figure 8**) correlated with culture endpoints such as CD27^+^CD38^+^ plasmablast differentiation and IgG, IgA, and IgM secretion in the culture supernatant. This analysis demonstrated that mainly the proportion of CD27^+^ cells at the start of culture correlated with differentiation into CD27^+^CD38^+^ cells on days 6 and 9 in both TD and TI stimuli (**Supplementary Tables 1**, **2**). Interestingly, the CD40L, IL-21, and IL-4 stimulation showed a negative correlation between the proportion of CD27^+^ cells and differentiation (**Supplementary Table 1**). This negative correlation was, however, again reversed when anti-BCR stimulation was also added. With regard to Ig-specific secretion, the addition of IL-4 positively correlated with day 6 IgG concentrations but negatively correlated with day 9 IgG concentrations. In TI conditions, the proportion of CD27^+^IgG^+^ and CD27^+^IgM^−^ cells positively correlated with CD27^+^CD38^+^ differentiation in all combinations of CpG, IL-2, BAFF, and anti-BCR stimuli, indicating that these subsets undergo more efficient differentiation in response to these stimuli compared to other subsets. With regard to Ig-specific secretion, a higher proportion of CD27^−^ cells at the start of culture negatively correlated with IgG and IgA secretion. Taken together, the proportion of CD27^+^ and isotype-specific subsets significantly determined the result of TD and TI cultures.

### Preserved *In-Vitro* B-Cell Differentiation in Cryopreserved PBMCs

**Figure 5:**
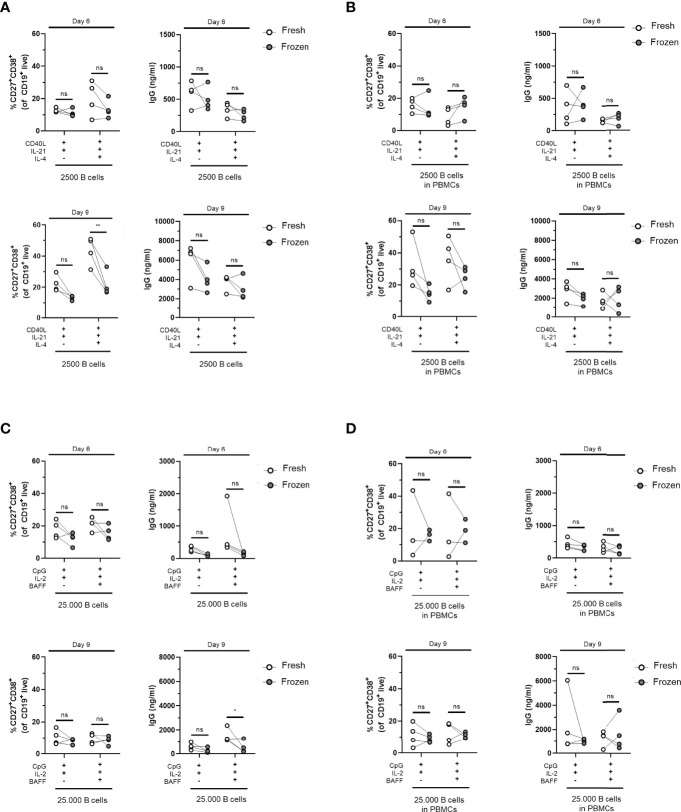
Cryopreserved B cells respond similarly to freshly isolated cells in T-cell-dependent and T-cell-independent assays. Total human B cells were isolated from fresh PBMCs (indicated in white) or frozen PBMCs (indicated in gray) and cultured for 6 and 9 days (*n* = 4). **(A, B)** Using T-cell-dependent (TD) stimuli (CD40L and IL-21 with/without IL-4), 2,500 B cells (fresh and frozen) were cultured under conditions described previously **(A)** without PBMCs (condition II) and **(B)** as PBMC culture (condition II.2). Frequencies of CD27^+^CD38^+^ B cells (left panel) and IgG production (right panel) on day 6 (upper graphs) and day 9 (lower graphs) are shown. **(C, D)** Using T-cell-independent (TI) stimuli (CpG and IL-2 with/without BAFF), 25,000 B cells (fresh and frozen) were cultured under conditions described previously **(C)** without PBMCs (condition IV) and **(D)** with PBMCs (condition IV.2). Frequencies of CD27^+^CD38^+^ B cells (left panel) and IgG production (right panel) on day 6 (upper graphs) and day 9 (lower graphs) are shown. Each data point represents the mean of an individual donor with duplicate culture measurements. Mean values are represented by bars and the error bars depict SEM. *P*-values were calculated using two-way ANOVA with Sidak’s multiple comparison test. *P ≤ 0.05, **P ≤ 0.01. ns, not significant.

The decision to use fresh PBMCs or cryopreserved PBMCs for an assay or study will depend on the assay itself as well as the logistics of handling samples. The collection of patient samples often involved the freezing of samples; therefore, the effect of freezing and thawing was assessed on the B-cell differentiation potential in our optimized TD and TI assays. For this purpose, the B-cell differentiation experiments were repeated on frozen samples of previously used healthy donors, either total PBMCs or isolated CD19^+^ B cells from thawed PBMCs, and assessed their B-cell proliferation and differentiation potential by plasmablast formation using FACS and Ig secretion. Interestingly, the CD19^+^ counts showed an increase in TD-stimulated frozen samples compared to fresh samples, although this difference was not significant (**Supplementary Figures 9A, B**). These trends were less clear in TI-stimulated frozen samples (**Supplementary Figures 9C, D**). Using the culturing conditions II and II.2 described above with CD40L and IL-21 stimulation, with or without IL-4, we detected a tendency to generate less CD27^+^CD38^+^ plasmablasts and subsequently lower secretion of IgG, IgA, and IgM in the supernatants of frozen samples after 6 and 9 days of culturing in all conditions tested, although we found no significant difference using the preferred CD40L and IL-21 stimulation (**Figures 5A, B** and **Supplementary Figures 10A, B**). Using the aforementioned conditions IV and IV.2 starting with 25,000 B cells and CpG and IL-2 stimulation supplemented with or without BAFF, again, we observed lower percentages of CD27^+^CD38^+^ plasmablasts and IgG, IgA, and IgM secretion in the culture supernatants after 6 and 9 days of culturing when compared to their matched fresh sample (**Figures 5C, D** and **Supplementary Figures 10C, D**). Thus, B cells obtained from cryopreserved PBMCs retained their ability to differentiate after *in-vitro* culturing using TD and TI stimulation although we observed a small decrease in their differentiation potential.

## Discussion

In this study, we report the optimized and efficient protocols for *in-vitro* B-cell differentiation using both TD and TI stimulation while requiring very low numbers of B cells. This has been accomplished by comparing several factors essential for optimal expansion, proliferation, and differentiation of B cells, including stimulation duration, seeding density, and combinations of activating stimuli reported in various publications for *in-vitro* B-cell differentiation. Here, we provide a one-step culture system starting with isolated CD19^+^ B cells or PBMCs corrected for B-cell counts. We demonstrate the successful generation of plasmablasts and plasma cells by measuring different parameters, including phenotypic markers (CD27, CD38, and CD138) combined with functional characteristics (IgG, IgA, and IgM secretion). Despite the small decrease in differentiation efficiency when using cryopreserved samples, there are numerous reasons why using frozen PBMCs is favored over fresh samples. The two main reasons are as follows: patient sampling is often done in outpatient clinics that are not in close proximity to laboratory facilities where cellular assays are performed; secondly, patient cohorts are often sampled longitudinally, and to prevent assay-to-assay variation, samples are stored for prolonged periods of time and thawed simultaneously. The loss in assay sensitivity with regard to differentiation may be minimized by narrowing down the time span in which all samples are handled or by taking along a known control. However, it should be noted that controls are preferentially also frozen PBMCs and handled in a comparable manner as the patient samples. The low number of required B cells determined here is ideal as patient samples are scarce and have value for multiple immunological assays. We believe that the conclusions and recommendations from this study will provide a basis for optimized protocols that can be used to study patient-related differences among patient cohorts of B-cell-mediated diseases and to screen compounds that target B-cell differentiation.

To date, a plethora of different conditions for inducing B-cell differentiation has been published (summarized in **Table 1**). The strength of the current study is the inclusion and comparison of many variables and different stimuli. However, due to study size limitations, it was not possible to include and compare all previously reported stimuli. The chosen reference stimuli for the TD assay, CD40L and IL-21, mimic the *in-vivo* activation and differentiation in germinal centers (GCs), where B cells interact with CD40L- and IL-21-expressing follicular T helper cells (1, 32). In our experience, CD40L-expressing fibroblasts are the strongest activators of B cells by providing sufficient CD40 binding and crosslinking (data not shown). Interestingly, in the assay of both B cells and PBMCs, different kinetics of B-cell expansion, proliferation, and Ig secretion were observed when the ratio of starting B cell to 3T3-CD40L feeder cell was increased. Specifically, in conditions III and III.2, starting with only 250 B cells, very high levels of IgM (but limited IgG) were observed on day 9, coinciding with an increased percentage of B cells with a CD27^−^CD38^+^ transitional-like phenotype. It is important to note that comparing the three conditions in terms of absolute Ig production during the 6- and 9-day culture period has its limitations as the number of B cells during these culture periods differed to a great extent and could influence Ig secretion (**Figure 1B** and **Supplementary Figure 2B**). In our data, higher availability of CD40L resulted in increased expansion of B cells and, despite cell number differences, a higher ratio of secreted IgM compared to IgA and IgG. As many facets of B-cell differentiation are linked to cell division, it is possible that the timing of isotype switching or the outgrowth of specific B-cell subsets occurs differently in these culture settings. Since we studied B-cell differentiation in bulk B cells and PBMCs, the specific effects of CD40L on naive B cells, IgM^+^ memory cells, or isotype-switched memory cells cannot be distinguished. These subsets, however, have been shown to have different requirements for stimulation with regard to differentiation into antibody-secreting cells (33, 34). Although we cannot make firm conclusions based on our findings, our data suggest and are in agreement with previous data that CD40 co-stimulation together with IL-21 regulates B-cell differentiation and Ig production and that these read-outs are driven by CD40L availability (17). However, we acknowledge that the effect of media exhaustion could also play a role in these cultures. Refreshing the culture media could lead to increased differentiation and Ig secretion, specifically in cultures with higher starting cell numbers. This was not opted for in the current study as we sought to maintain a one-step, easy, and simple culture to be used in clinical studies. Concluding from our data, we choose 2,500 B cells as the optimal starting number so as not to preferentially outgrow specific subpopulations or isotypes. In order to mimic the *in-vivo* response to cognate antigen more closely, we added an anti-BCR trigger (35). However, we show that this hampers Ig detection. This has to be taken into account when these assays are applied for specific research questions where omitting an anti-BCR trigger is not desirable. The same holds true for adding IL-4 to the assays. Previously, we have shown that IL-4 addition is beneficial for B-cell differentiation of naive B cells but only in circumstances with low CD40L stimulation (17). In accordance with other studies using total CD19^+^ B cells, we show that continuous IL-4 in our assay hampers Ig secretion compared to CD40L and IL-21 alone, indicating a lack of commitment to antibody secretion (5). Because of this, we do not recommend using IL-4 when investigating the induction of plasmablast differentiation. However, as IL-4 plays a major role regarding pre-GC B-cell priming and promotes class switch to IgG4 and IgE, studies focusing on, e.g., IgG4-related disease or allergy, might want to use IL-4 nonetheless. IgE and IgG subclass production was, however, not determined in the cultures presented here.

To mimic *in-vivo* TI responses, the most commonly used stimulation is TLR-9 activation through CpG, mimicking antigen activation (**Table 1**). Activation with CpG induces the proliferation of both human naive and memory B cells (data not shown), while the differentiation of naive B cells is only observed in cultures where PBMCs are present or T-cell-derived cytokines such as IL-2 are supplemented (16, 18). Adding to this, *in-vivo* TI stimulation has been shown to result in long-lived plasma cell generation (14). This together indicates that though direct T–B interactions may not be required, a supportive microenvironment may be crucial to gain plasmablast fate and sustain plasma cell generation in TI responses *in-vitro*. Condition IV with 25,000 starting B cells was identified as a minimum when stimulating isolated CD19^+^ B cells with CpG and IL-2 due to limited B-cell survival in this culture. Considering the significant decrease of CD19^+^ B cells between day 6 and day 9 in condition IV, we do not recommend culturing longer than 6 days, although higher amounts of immunoglobulins can be measured with a longer culture period. Restimulation of cells can be opted for, but this was not investigated in this study. Finally, compared to isolated B-cell cultures, PBMC TI cultures showed better survival of B cells (conditions IV and V versus conditions IV.2 and V.2) (**Table 3**). Although the microenvironment provided by PBMCs may support survival, there was no observation of increased differentiation.

BAFF protein is expressed by myeloid lineage cells and acts as both cell surface-associated and soluble forms (36, 37). BAFF has been shown to activate class switch recombination in human B cells, which can be enhanced by BCR cross-linking (38). Ever since, research groups have used BAFF in B-cell differentiation assays but most frequently in combinatorial use with CD40 stimulation, preventing the dissection of their individual effects. In the current study, limited effects of BAFF, in addition to anti-BCR stimulation, were found on plasmablast formation. Interestingly, a donor-dependent effect of BAFF stimulation on the Ig secretion was observed. Healthy donors and patients possibly differ in their expression of BAFF-responding receptors at baseline. Furthermore, because activated monocytes and T cells can also express BAFF-responding receptors, it raises the possibility that in the PBMC cultures, the addition of BAFF will stimulate the rest of the PBMCs rather than the B cells. Finally, as monocytes are known to increase BAFF secretion upon TLR-9 stimulation, it is possible that these cells were already supplying sufficient BAFF to the B cells within the PBMC TI cultures (29). Altogether, although no detrimental effects of BAFF on B-cell differentiation were observed in our assay, using BAFF should be complemented with appropriate analysis of its compliant receptors at baseline and throughout the assay.

The data presented here show that for the TD condition, stimulating as little as 2,500 CD19^+^ B cells with CD40L and IL-21 results in significant expansion, differentiation, and secretion of IgM, IgA, and IgG. For specific purposes, even lower cell numbers can be used. Interestingly, IL-4 did not affect differentiation but did significantly reduce antibody secretion. For studying TI responses, stimulating 25,000 CD19^+^ B cells with CpG and IL-2 results in proliferation, differentiation, and IgM, IgA, and IgG production. We do not recommend using lower cell numbers for this condition. Interestingly, the addition of BAFF resulted in significant increases in IgM, IgA, and IgG production in TI CD19^+^ B-cell cultures. However, this effect is absent in PBMC cultures. Furthermore, we show that both these protocols can be performed with PBMC cultures, omitting the need for B-cell isolation and, thus, making them highly suitable for clinical research. We do, however, recommend that B-cell numbers are corrected using measured B-cell percentages, after thawing, as these percentages are variable between donors. Furthermore, monocyte and T-cell involvement in these cultures could not entirely be excluded possibly explaining mixed results when comparing this and previous studies (16, 18). In addition, we recommend determining the B-cell subset and Ig-isotype composition in (patient) samples before culture as the proportion of CD27^+^ and class-switched cells correlates with differentiation and Ig secretion results. As healthy donor cells were used here, it will be interesting to monitor, for example, (longitudinal) autoimmune patient sample composition to determine if the same correlations are present and if these are affected during immunosuppressive treatment. Finally, we acknowledge that for specific research questions or patient samples, it will be worthwhile to study additional B-cell functions (e.g., pro- and anti-inflammatory cytokine production) (39) or to sort B-cell subsets like naive (17), memory, or MZ B cells (40, 41) or IgG4-expressing cells (42). It should be taken into consideration, however, how much material is available and how much material will be lost upon sorting and if, instead, it is possible to investigate the subset of interest within PBMC cultures.

In conclusion, it is still an active area of investigation to define how autonomous factors control TD and TI responses in healthy donors or patients with B-cell-mediated diseases. Future research is needed to define these autonomous factors and address signaling pathways involved in both beneficial and unwanted plasma cell development. Comparing patients and healthy donors in optimized cultures and assays that detect gene expression and post-translational modifications such as phosphorylation or ubiquitination by intracellular staining methods (43) may aid in these research questions. The TD and TI assays described here in condition II (and II.2), being 2,500 CD19^+^ B cells stimulated with CD40L and IL-21, and in condition IV (and IV.2), being 25,000 CD19^+^ B cells stimulated with CpG, IL-2, and possibly BAFF, support the efficient differentiation of human primary B cells into plasma cells, with warranted B-cell expansion, proliferation, and quantifiable production of IgG, IgA, and IgM. Due to the minimalistic nature of the protocols, the results from different labs and facilities will be highly comparable. These assays will allow in-depth dissection of B-cell differentiation pathways in B cells of healthy individuals and patients.

## Data Availability Statement

The raw data supporting the conclusions of this article will be made available by the authors, without undue reservation.

## Ethics Statement

The studies involving human participants were reviewed and approved by The Medical Ethics Committee of Sanquin Blood Supply, The Netherlands. The patients/participants provided their written informed consent to participate in this study.

## Author Contributions

JK, CM, and DV designed the experiments. CM and DV performed the experiments and analyzed the data. TR and AB critically revised the manuscript. SH and TK devised the concept, supervised data interpretation, and critically revised the manuscript. The manuscript was revised and approved by all authors.

## Funding

This collaboration project is financed by the PPP Allowance made available by Top Sector Life Sciences & Health to Samenwerkende Gezondheidsfondsen (SGF) under project number LSHM18055-SGF to stimulate public–private partnerships and co-financed by health foundations that are part of the SGF. This project was also funded by the Landsteiner Foundation for Blood Transfusion Research (project grant number: LSBR 1609) and Sanquin Product and Process Development Call 2020.

## Conflict of Interest

The authors declare that this research was conducted in the absence of commercial or financial relationships that could be construed as a potential conflict of interest. The handling editor JG declared a shared affiliation with the authors DV and TK.

## Publisher’s Note

All claims expressed in this article are solely those of the authors and do not necessarily represent those of their affiliated organizations, or those of the publisher, the editors and the reviewers. Any product that may be evaluated in this article, or claim that may be made by its manufacturer, is not guaranteed or endorsed by the publisher.
