# Supplementary material for: Optimized Protocols for In-Vitro T-Cell-Dependent and T-Cell-Independent Activation for B-Cell Differentiation Studies Using Limited Cells
Source: Front Immunol. 2022 Jun 29;13:815449. doi: 10.3389/fimmu.2022.815449 (PMC9278277; doi:10.3389/fimmu.2022.815449)
Supplement: Supplementary file 1 [file DataSheet_1.docx]

Supplementary Material

**Optimized protocols for in vitro T cell-dependent and T cell-independent activation for B cell differentiation studies using limited cells**

Marsman, Verhoeven et al.

**
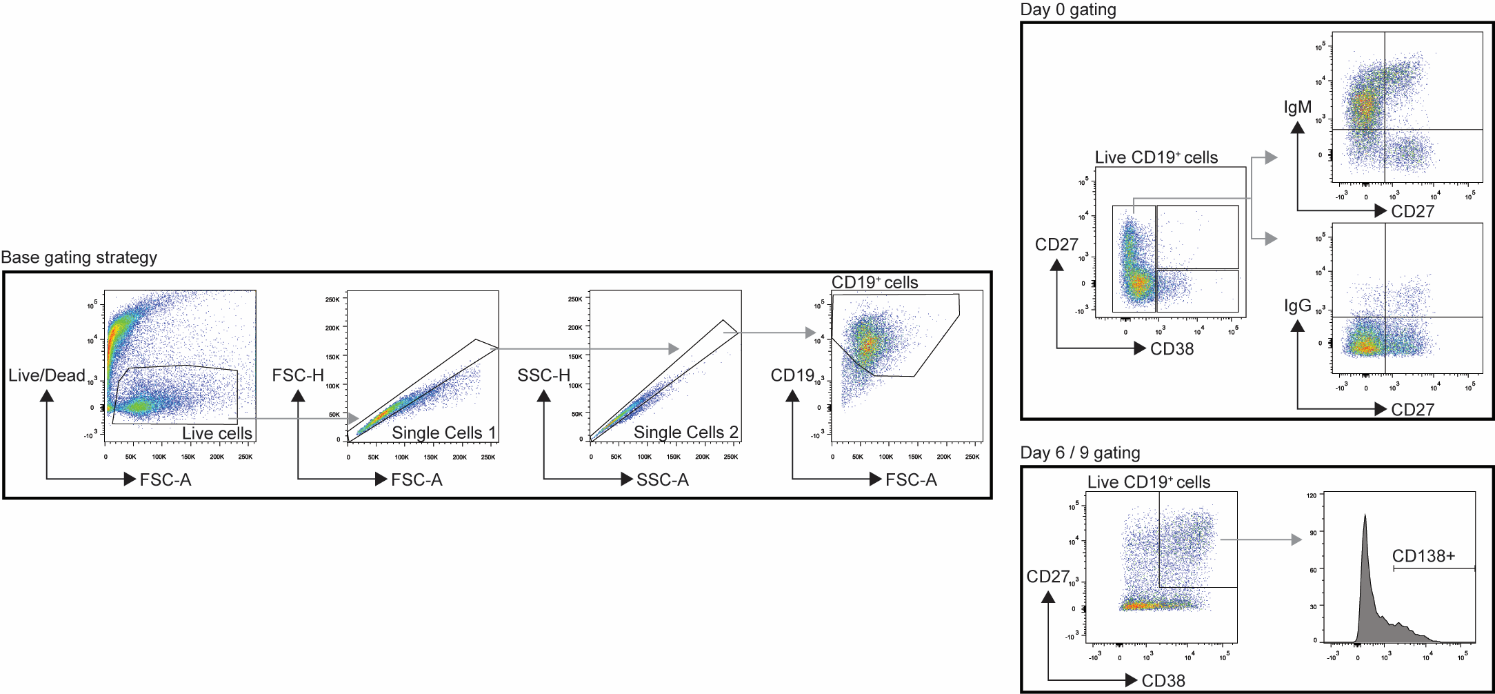
**

**Supplemental Figure 1. Flow cytometry gating strategy.**Total lymphocytes were first gated on a forward scatter (FSC-A) versus Live/Dead plot and then gated on single cells using both FSC-H/FSC-A and SSC-H/SSC-A plots. Next, CD19^+^ B cells were gated and subsets of interest were gated within the CD19^+^ B cells. On day 0 the initial characterization of the B cell compartment was performed, including pre-existing CD27^+^ CD38^+^ plasmablasts, CD27^-^ CD38^+^ and CD27^+/-^ CD38^-^ B cells. IgM and IgG expression within the CD27^+/-^ CD38^-^ B cells was used to determine the naïve, non-switched memory and switched memory population at baseline. On day 6 and day 9 CD27^+^ CD38^+^ plasmablasts and CD27^+^ CD38^+^ CD138^+^ plasma cells were analyzed.

**
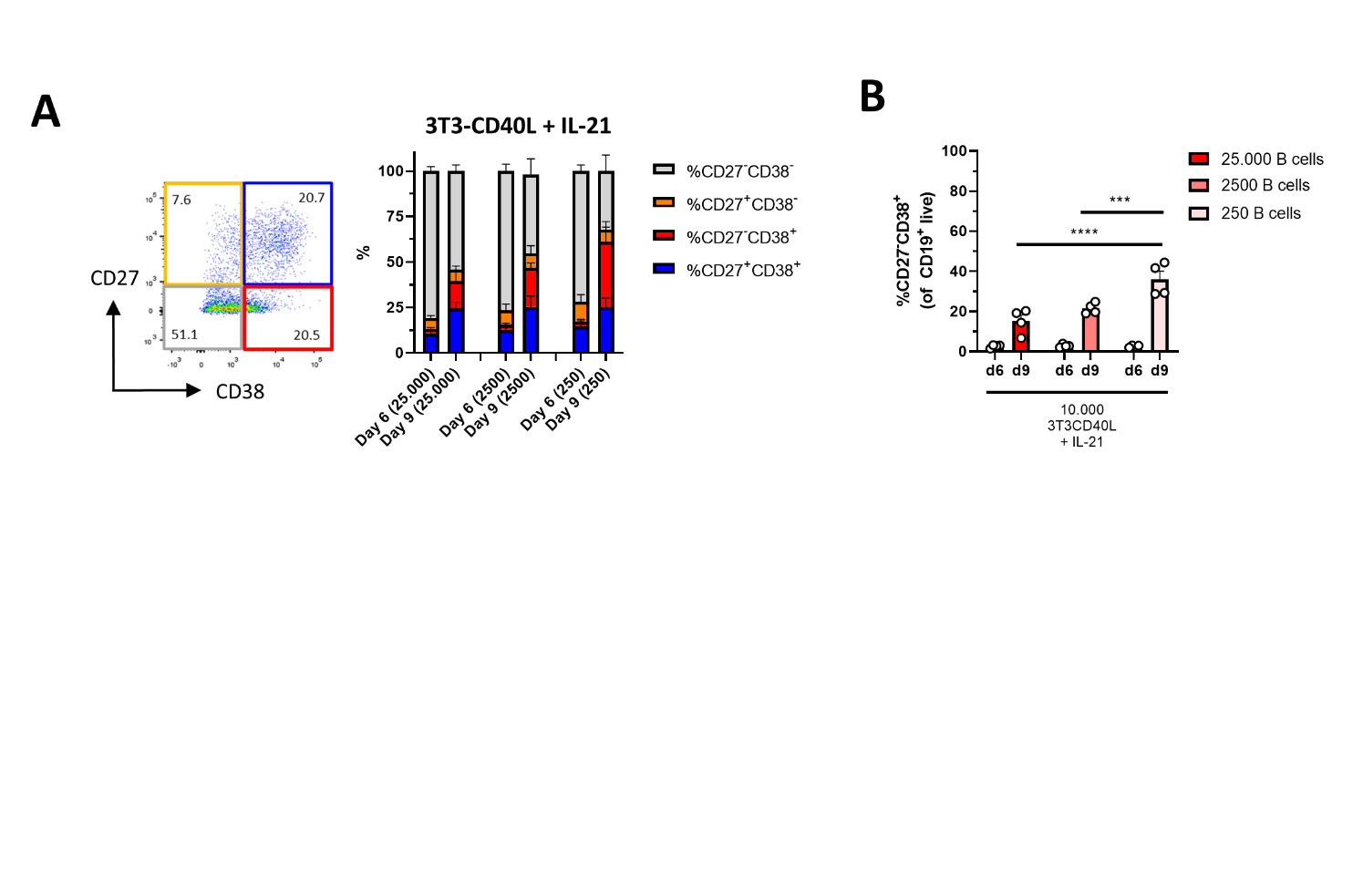
**

**Supplemental Figure 2. CD27 and CD38 expression of stimulated primary human B cells.
(A)** Representative FACS plot (left panel) show gating strategy of CD27/CD38 subpopulations and quantification of the relative percentages of CD27 and CD38 subpopulations in the total CD19^+^ B cell population between 6 and 9 days of culture (n = 4). **(B)** The frequency of CD27^-^CD38^+^ B cells. Each data point represents the mean of an individual donor with duplicate culture measurements. Mean values are represented by bars and the error bars depict SEM. P values were calculated using two-way ANOVA with Tukey’s multiple comparison test. *** P ≤ 0.001, **** P ≤ 0.0001.

**
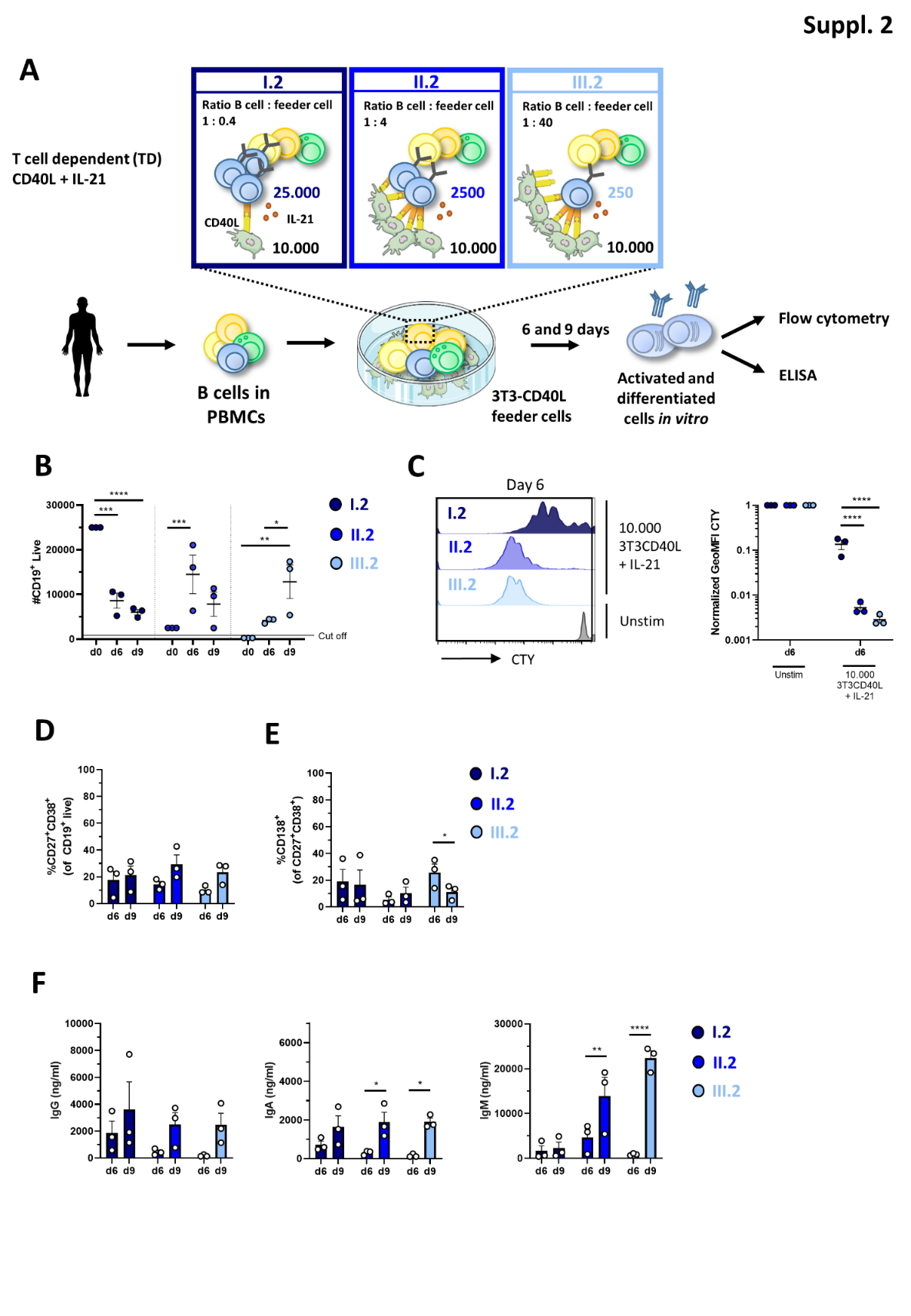
**

**Supplemental Figure 3. Proliferation, differentiation and antibody production of primary human CD19^+^ B cells after T cell dependent in vitro stimulation and culturing of PBMCs.
(A)** Schematic overview of the T cell dependent (TD) culture system to induce B cell differentiation. A total of 25000, 2500 or 250 CD19^+^ human B cells in PBMCs (n = 3) were stimulated with a human-CD40L-expressing 3T3 feeder layer and recombinant IL-21 (50 ng/mL) enabling condition I.2 (dark blue), II.2 (cobalt blue) and III.2 (light blue). Cells were analyzed at day 6 and day 9 by flow cytometry to evaluate plasmablast and plasma cell generation. The supernatant was collected at day 6 and day 9 to evaluate IgG, IgA and IgM production by ELISA. **(B)** The number of live CD19^+^ events was analyzed using flow cytometry. A cut off of 1000 events was used to proceed with further analysis. **(C)** Representative histograms of CTY dilution (left panel) and quantification (right panel) on day 6 compared to their unstimulated condition. **(D)** The frequency of CD27^+^CD38^+^ B cells and **(E)** CD27^+^CD38^+^CD138^+^ B cells was analyzed by using flow cytometry. **(F)** IgG, IgA and IgM production in culture supernatants was evaluated by ELISA after 6 and 9 days (n = 3). Each data point represents the mean of an individual donor with duplicate culture measurements. Mean values are represented by bars and the error bars depict SEM. P values were calculated using two-way ANOVA with Sidak’s multiple comparison test. * P ≤ 0.05, ** P ≤ 0.01, *** P ≤ 0.001, **** P ≤ 0.0001.


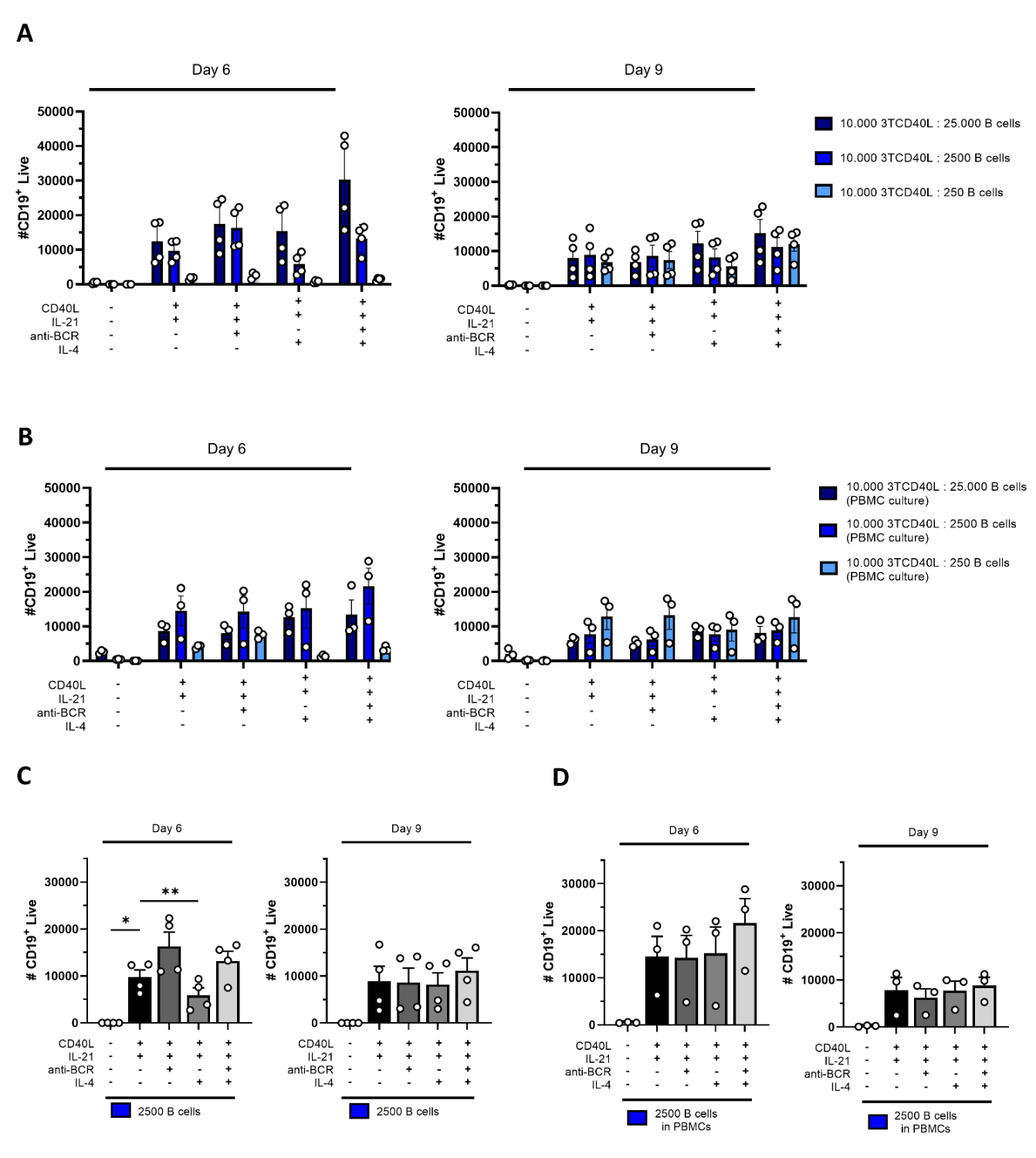


**Supplemental Figure 4. CD19^+^ live cells in TD cultures with anti-BCR and IL-4.
(A – B)** Frequencies of CD19^+^ live cells on day 6 and day 9 in conditions described in Figure 1A (condition I, II, III) and Suppl. Fig. 3A (condition I.2, II.2, III.2) (PBMC cultures) with or without anti-BCR (anti-Ig F(ab)2 mix (5 µg/mL) targeting IgM, IgG and IgA) and/or recombinant IL-4 (25 ng/mL). Frequencies of CD19^+^ live cells on day 6 and day 9 in **(C)** condition II (n=4) and **(D)** condition II.2 (n = 3) including statistics. Each data point represents the mean of an individual donor with duplicate culture measurements. Mean values are represented by bars and the error bars depict SEM. P values were calculated using two-way ANOVA with Sidak’s multiple comparison test. * P ≤ 0.05, ** P ≤ 0.01.

**
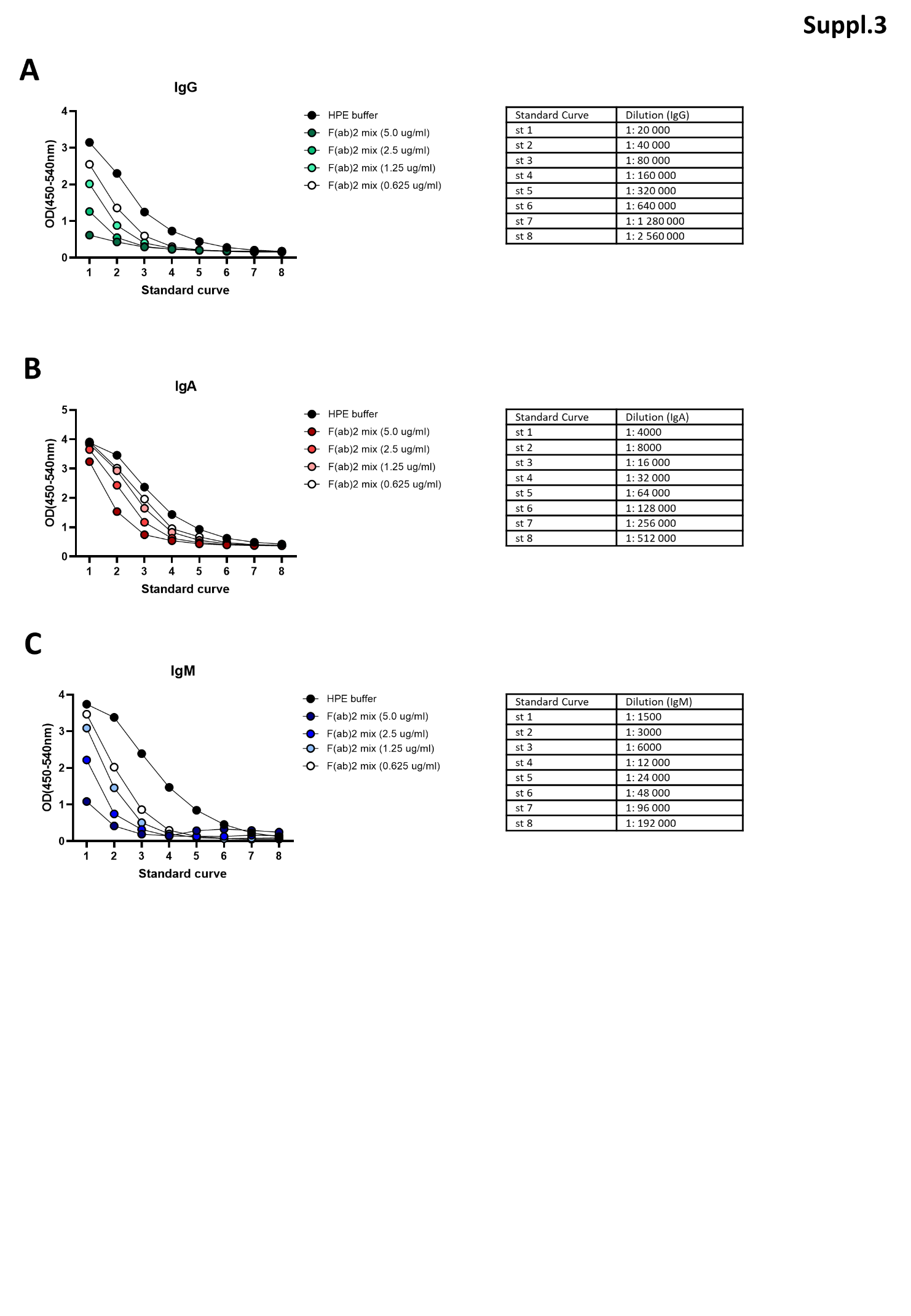
**

**Supplemental Figure 5. Anti-IgA/G/M F(ab’)_2_ fragments interfere with ELISA readouts.**Interference of F(ab’)2 fragment Goat Anti-Human IgA/G/M in **(A)** IgG, **(B)** IgA and **(C)** IgM ELISA. Serial dilutions of F(ab’)2 fragments (5, 2.5, 1.25 and 0.625 μg/mL) were added to the standard curve dilutions as indicated. Black lines indicate no F(ab’)2 fragments added.

**
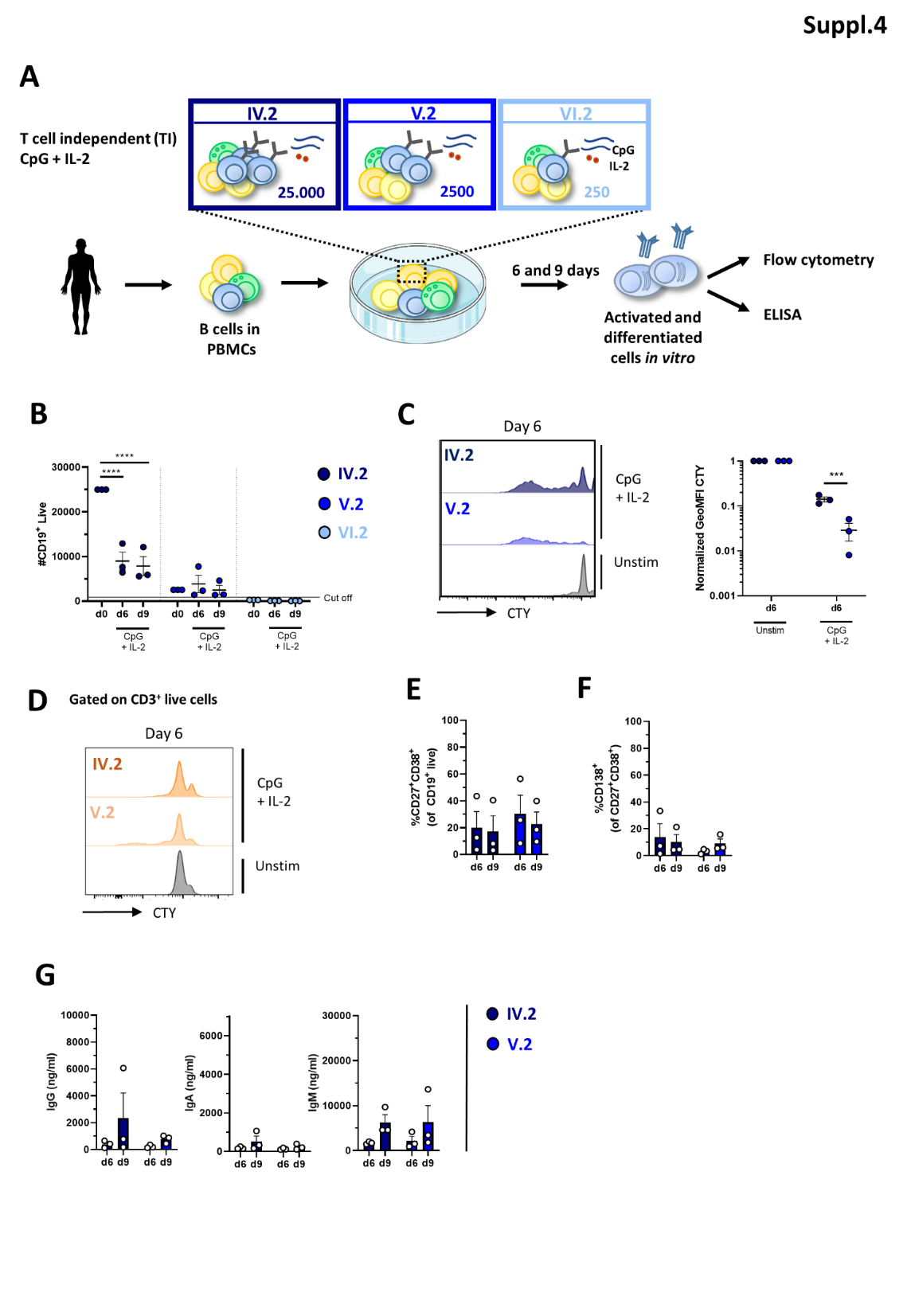
**

**Supplemental Figure 6. Proliferation, differentiation and antibody production primary human CD19^+^ B cells after T cell independent in vitro stimulation and culturing of PBMCs.
(A)** Schematic overview of the T cell independent (TI) culture system to induce B cell differentiation. A total of 25000, 2500 or 250 CD19^+^ human B cells and PBMCs (n = 3) were stimulated with CpG (1 µM) and IL-2 (50 ng/ml) enabling condition IV.2 (dark blue), V.2 (cobalt blue) and VI.2 (light blue). Cells were analyzed at day 6 and day 9 by flow cytometry to evaluate plasmablast and plasma cell generation. The supernatant was collected at day 6 and day 9 to evaluate IgG, IgA and IgM production by ELISA. **(B)** The number of live CD19^+^ events was analyzed using flow cytometry. A cut off of 1000 events was used to proceed with further analysis. **(C)** Representative histogram of CTY dilution (left panel) and quantification (right panel) of condition IV.2 and V.2 on day 6 compared to their unstimulated condition. **(D)** Analysis of proliferation by CTY dilution of CD3^+^ T cells in condition IV.2 and V.2 on day 6. **(E)** The frequency of CD27^+^CD38^+^ B cells and **(F)** CD27^+^CD38^+^CD138^+^ B cells. **(G)** IgG, IgA and IgM production in culture supernatants was evaluated by ELISA after 6 and 9 days (n = 4). Each data point represents the mean of an individual donor with duplicate culture measurements. Mean values are represented by bars and the error bars depict SEM. P values were calculated using two-way ANOVA with Sidak’s multiple comparison test. * P ≤ 0.05, ** P ≤ 0.01, *** P ≤ 0.001, **** P ≤ 0.0001.

**
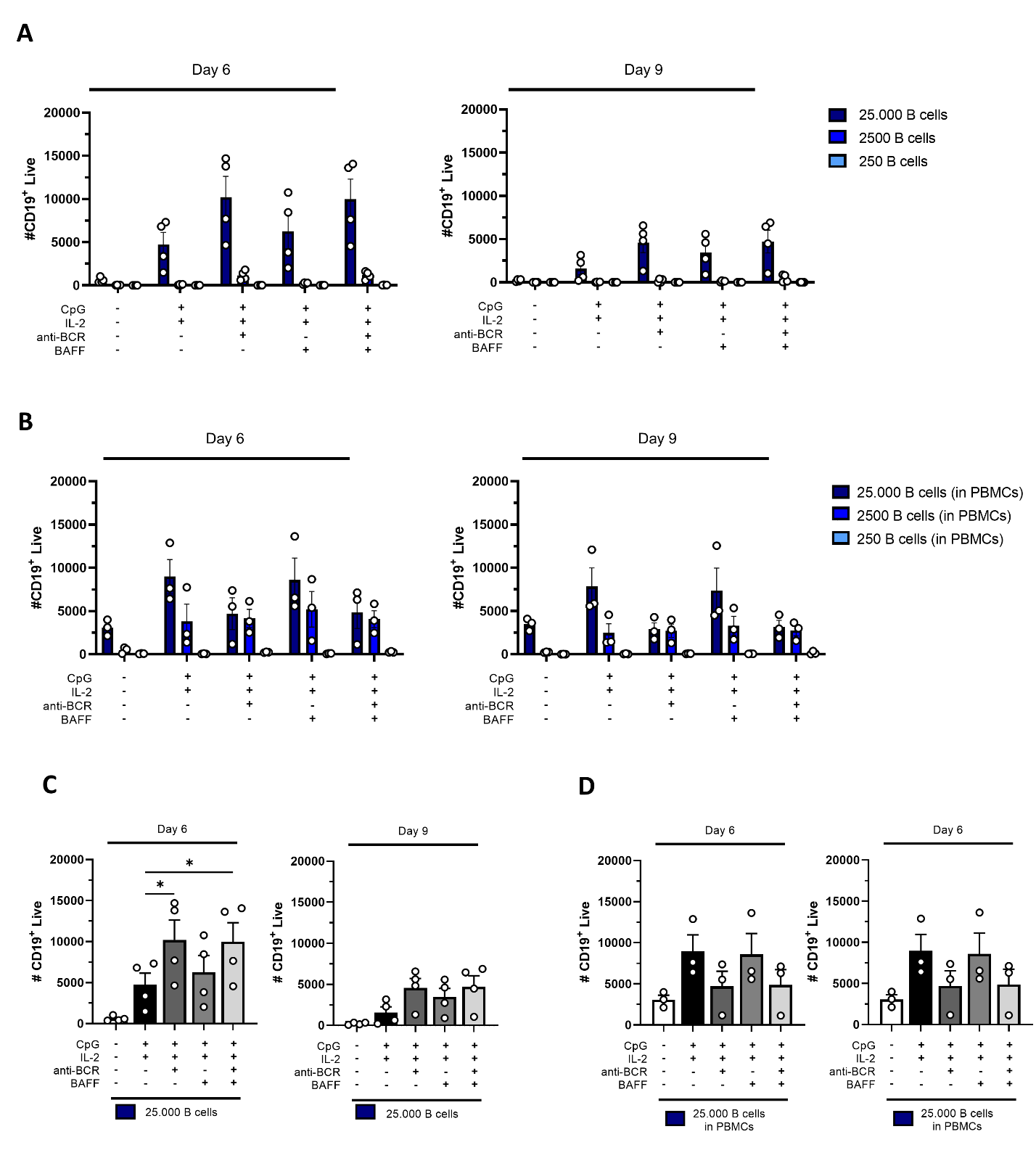
**

**Supplemental Figure 7. CD19^+^ live cells in TI cultures with anti-BCR and BAFF.
(A – B)** Frequencies of CD19^+^ live cells on day 6 and day 9 in conditions described in Figure 3A (condition IV, V, VI) and Suppl. Fig. 6A (condition IV.2, V.2, VI.2) (PBMC cultures) with or without anti-BCR (anti-Ig F(ab)2 mix (5 µg/mL) targeting IgM, IgG and IgA) and/or BAFF (100 ng/mL). Frequencies of CD19^+^ live cells on day 6 and day 9 in **(C)** condition IV (n=4) and **(D)** condition IV.2 (n = 3) including statistics. Each data point represents the mean of an individual donor with duplicate culture measurements. Mean values are represented by bars and the error bars depict SEM. P values were calculated using two-way ANOVA with Sidak’s multiple comparison test. * P ≤ 0.05.


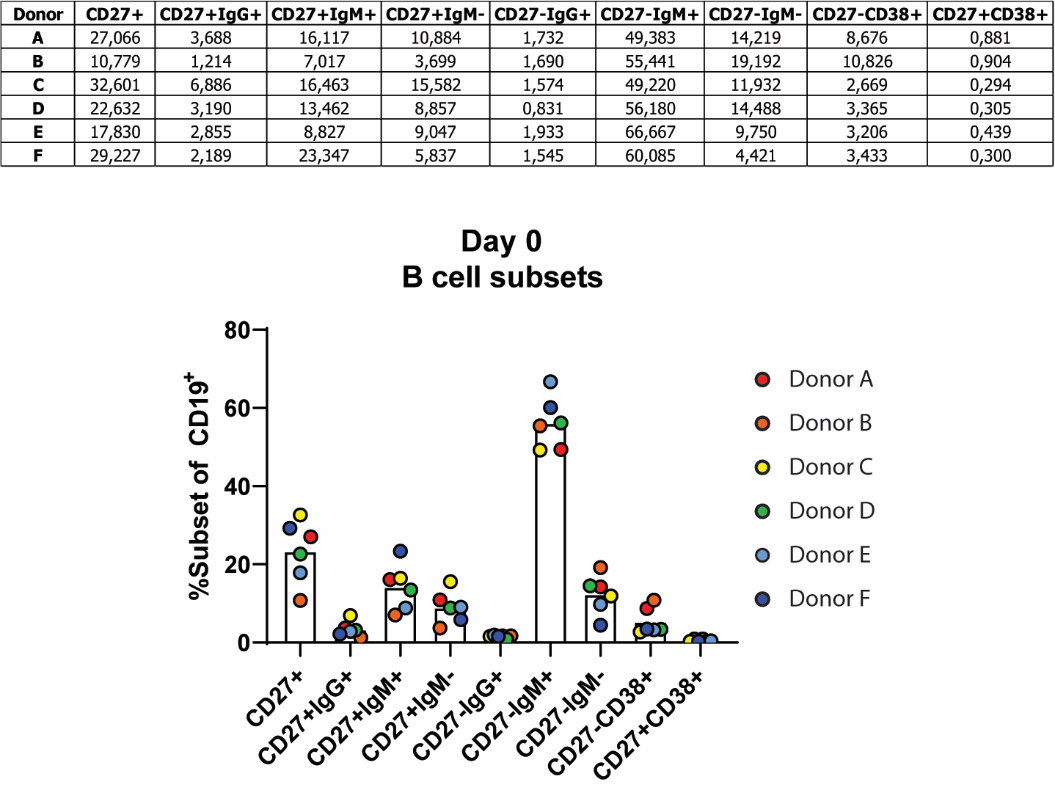


**Supplemental Figure 8. Characterization of the B cell compartment at day 0.**On day 0 the initial characterization of the B cell compartment of donor A-F was performed (for gating strategy see Suppl. Fig. 1), including pre-existing CD27^+^ CD38^+^ plasmablasts, CD27^-^ CD38^+^ and CD27^+/-^ B cells. IgM and IgG expression within the CD27^+/-^ CD38^-^ B cells was used to determine the naïve, non-switched memory and switched memory distribution at baseline. These baseline % were used for correlation analysis (see Suppl. Table 1 and 2).

,**
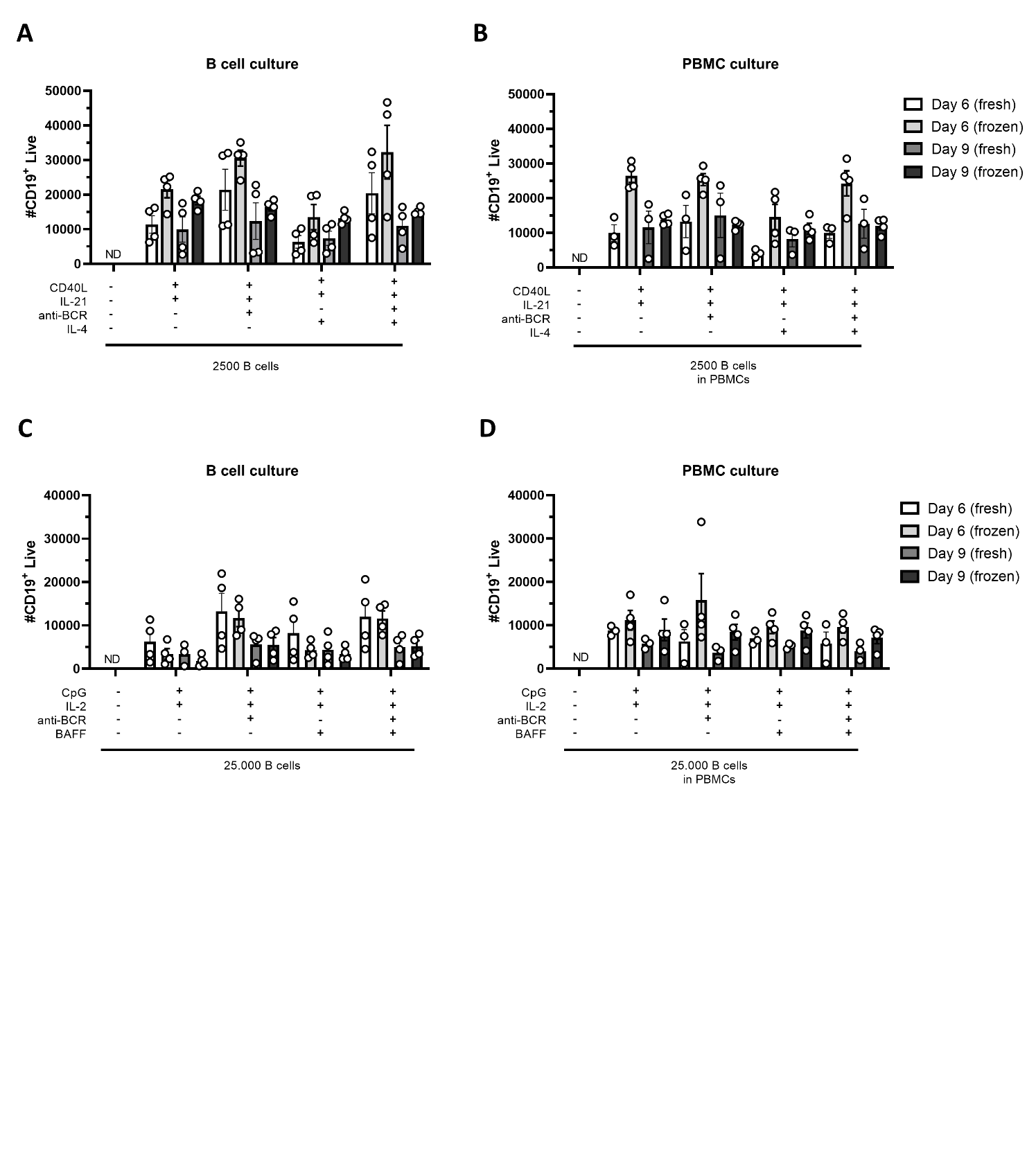
**

**Supplemental Figure 9. Frequencies of CD19^+^ live cells in TD and TI cultures using cryopreserved and freshly isolated B cells.**Frequencies of CD19^+^ live cells measured by flowcytometry from cultures with B cells isolated from fresh PBMCs or frozen PBMCs which where cultured for 6 and 9 **(A-B)** with TD stimuli with and without PBMCs (condition II and II.2) or **(C-D)** with TI stimuli (condition IV and IV.2). ND = not determined.

**
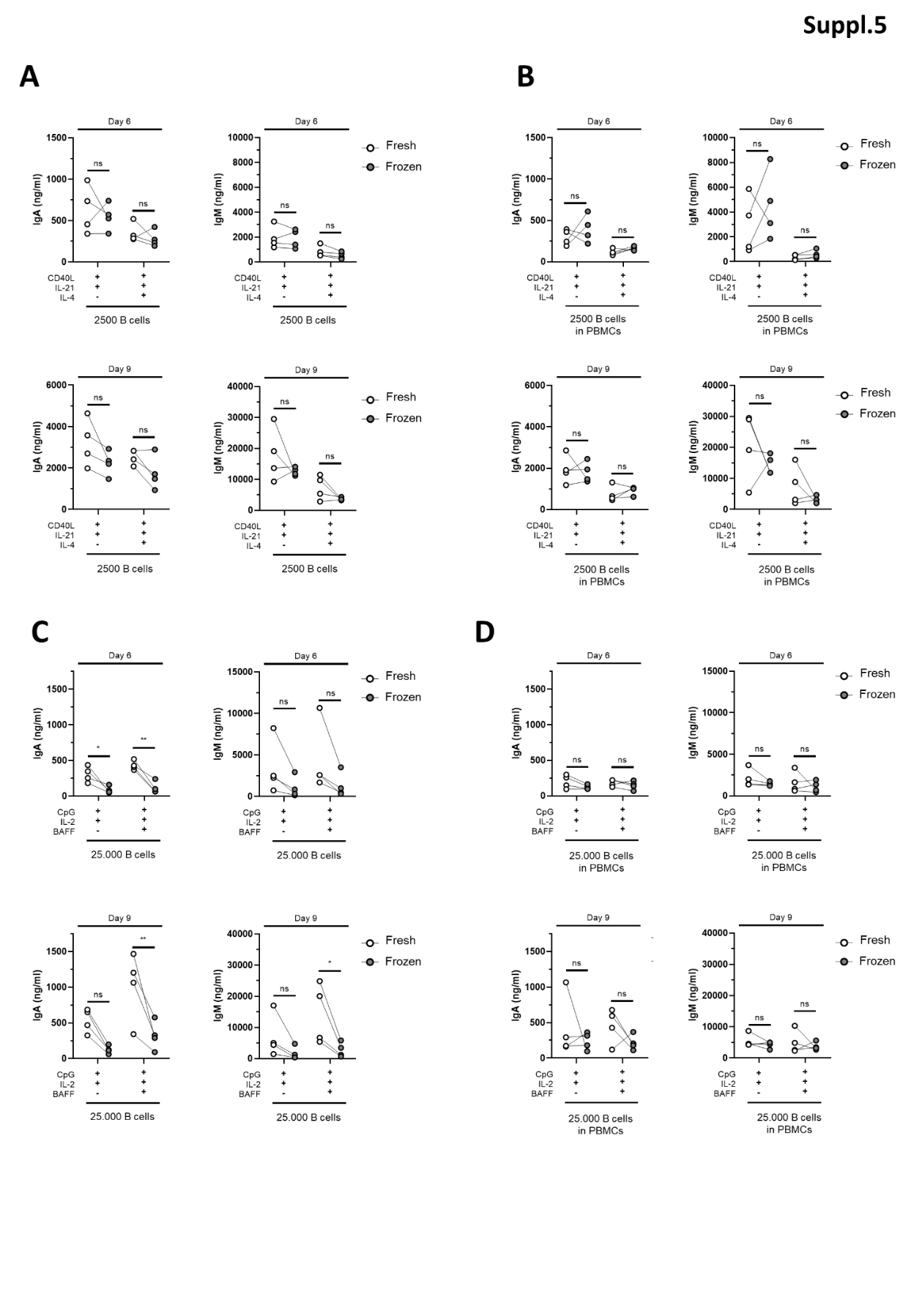
**

**Supplemental Figure 10. Cryopreserved and freshly isolated B cells produce similar amounts of antibodies in T cell dependent and independent assays.**Comparison of IgA and IgM production of B cells isolated freshly from PBMCs or from cryopreserved PBMCs obtained from the same healthy donor (n=4). Total human B cells were isolated from fresh PBMCs (indicated in white) or frozen PBMCs (indicated in gray) and cultured for 6 and 9 days. **(A-B)** Using T cell dependent (TD) stimuli (CD40L and IL-21 with/without IL-4) 2500 B cells (fresh and frozen) were cultured under conditions described previously **(A)** without PBMCs (condition II) and **(B)** with PBMCs (condition II.2). IgA (left panel) and IgM production (right panel) on day 6 (upper graphs) and day 9 (lower graphs) are shown. **(C-D)** Using T cell independent (TI) stimuli (CpG and IL-2 with/without BAFF) 25.000 B cells (fresh and frozen) were cultured under conditions described previously **(C)** without PBMCs (condition IV) and **(D)** with PBMCs (condition IV.2). IgA (left panel) and IgM production (right panel) on day 6 (upper graphs) and day 9 (lower graphs) are shown. Each data point represents the mean of an individual donor with duplicate culture measurements. Mean values are represented by bars and the error bars depict SEM. P values were calculated using two-way ANOVA with Sidak’s multiple comparison test. * P ≤ 0.05, ** P ≤ 0.01.
