# Supplementary material for: Optimized Protocols for In-Vitro T-Cell-Dependent and T-Cell-Independent Activation for B-Cell Differentiation Studies Using Limited Cells
Source: Front Immunol. 2022 Jun 29;13:815449. doi: 10.3389/fimmu.2022.815449 (PMC9278277; doi:10.3389/fimmu.2022.815449)
Supplement: Supplementary file 2 [file DataSheet_2.docx]

**T2B consortium members**

PhD/Postdocs

Name Institute

Mrs. Annabel Ruiter LUMC

Mrs. Linda van der Weele AMC

Mrs. Karoline Kielbassa AMC

Mrs. Mariateresa Coppola VUMC

Mrs. Dorit Verhoeven AMC

Mrs. Jyaysi Desai LUMC

Mrs. Mirjam van der Burg LUMC

Mrs. Esther Vletter LUMC

Mrs. Maaike Braham RIVM

Mr. Matthias Busch MUMC

Mr. Carlo Bonasia UMCG

Mrs. Elisabeth Raveling UMCG

Mrs. Ruth Huizinga ErasmusMC

Mr. Niels Verstegen Sanquin

Mr. Casper Marsman Sanquin

Mrs. Sabrina Pollastro Sanquin

Mr. Koos van Dam AMC

Mr. Laurent Paardekooper LUMC

Mrs. Renée Ysermans MUMC

Mrs. Odilia Corneth ErasmusMC

Mrs. Annemarie Buisman RIVM

Mr. Rob van Binnendijk (RIVM)

Mrs Pauline van Schouwenburg (LUMC)

Mr. Marvyn Koning LUMC

Mr. Luuk Wieske AMC

Fundamental partners (Groupleaders / division heads)

Name Institute

Dr. Lisa van Baarsen AMC

Prof. dr. Nico Bos UMCG

Dr. Anja ten Brinke Sanquin

Dr. Eric Eldering AMC

Dr Cecile van Els RIVM

Prof. dr. Marieke van Ham Sanquin

Prof. dr. Peter Heeringa UMCG

Prof. dr. Rudi Hendriks ErasmusMC

Dr. Maartje Huijbers LUMC

Dr. Ruth Huizinga ErasmusMC

Prof. dr. Reina Mebius VUMC

Dr. Theo Rispens Sanquin

Prof. dr. Rene Toes LUMC

Dr. Jelle de Wit RIVM

Dr. Jan Damoiseaux MUMC

Dr. Wayel Abdulahad UMCG

Clinical partners

Name Institute

Dr. Filip Eftimov AMC

Dr. Casper Franssen UMCG

Prof. dr. Jaap Groothoff AMC

Prof. dr. Bart Jacobs ErasmusMC

Dr. Barbara Horvath UMCG

Prof. dr. Arnon Kater AMC

Dr. Joep Killestein VUMC

Prof. dr. Taco Kuijpers AMC

Dr. Karina de Leeuw UMCG

Dr. L Oosten LUMC

Dr. Pieter van Paassen UMC Maastricht

Dr. Bram Rutgers UMCG

Dr. Uli Scherer LUMC

Dr. Maarten Titulaer ErasmusMC

Prof. dr. Jan Verschuuren LUMC

Dr. Niek de Vries AMC

Dr. Diane van der Woude LUMC

Dr. Josephine Vos AMC

Prof. dr. Hendrik Veelken LUMC
